# Association of variation in the sugarcane transcriptome with sugar content

**DOI:** 10.1186/s12864-017-4302-5

**Published:** 2017-11-25

**Authors:** Prathima P. Thirugnanasambandam, Nam V. Hoang, Agnelo Furtado, Frederick C. Botha, Robert J. Henry

**Affiliations:** 1 Queensland Alliance for Agriculture and Food Innovation, The University of Queensland, St. Lucia, QLD 4072 Australia; 2 ICAR - Sugarcane Breeding Institute, Coimbatore, Tamil Nadu India; 3 College of Agriculture and Forestry, Hue University, Hue, Vietnam; 4 Sugar Research Australia, Indooroopilly, QLD 4068 Australia; 5 The University of Queensland, Room 2.245, Level 2, The John Hay Building, Queensland Biosciences Precinct [#80], 306 Carmody Road, St Lucia, QLD 4072 Australia

**Keywords:** Sucrose, Transcriptome, High and low sugar genotypes, Sucrose genes, Sugarcane transcriptome

## Abstract

**Background:**

Sugarcane is a major crop of the tropics cultivated mainly for its high sucrose content. The crop is genetically less explored due to its complex polyploid genome. Sucrose synthesis and accumulation are complex processes influenced by physiological, biochemical and genetic factors, and the growth environment. The recent focus on the crop for fibre and biofuel has led to a renewed interest on understanding the molecular basis of sucrose and biomass traits. This transcriptome study aimed to identify genes that are associated with and differentially regulated during sucrose synthesis and accumulation in the mature stage of sugarcane. Patterns of gene expression in high and low sugar genotypes as well as mature and immature culm tissues were studied using RNA-Seq of culm transcriptomes.

**Results:**

In this study, 28 RNA-Seq libraries from 14 genotypes of sugarcane differing in their sucrose content were used for studying the transcriptional basis of sucrose accumulation. Differential gene expression studies were performed using SoGI (*Saccharum officinarum* Gene Index, 3.0), SAS (sugarcane assembled sequences) of sugarcane EST database (SUCEST) and SUGIT, a sugarcane Iso-Seq transcriptome database. In total, about 34,476 genes were found to be differentially expressed between high and low sugar genotypes with the SoGI database, 20,487 genes with the SAS database and 18,543 genes with the SUGIT database at FDR < 0.01, using the Baggerley’s test. Further, differential gene expression analyses were conducted between immature (top) and mature (bottom) tissues of the culm. The DEGs were functionally annotated using GO classification and the genes consistently associated with sucrose accumulation were identified.

**Conclusions:**

The large number of DEGs may be due to the large number of genes that influence sucrose content or are regulated by sucrose content. These results indicate that apart from being a primary metabolite and storage and transport sugar, sucrose may serve as a signalling molecule that regulates many aspects of growth and development in sugarcane. Further studies are needed to confirm if sucrose regulates the expression of the identified DEGs or vice versa. The DEGs identified in this study may lead to identification of genes/pathways regulating sucrose accumulation and/or regulated by sucrose levels in sugarcane. We propose identifying the master regulators of sucrose if any in the future.

**Electronic supplementary material:**

The online version of this article (10.1186/s12864-017-4302-5) contains supplementary material, which is available to authorized users.

## Background

Among the domesticated grasses, sugarcane and sweet sorghum have undergone extensive selection for high accumulation of sucrose that serves as the primary sources of sugars for human and animal consumption, as well as ethanol production for fuel [1].The maturing sugarcane culm represents both an economically important and physiologically interesting experimental system to study the dynamics of carbohydrate partitioning and metabolism associated with the accumulation of high concentrations of sucrose. A distinctive feature of sugarcane is that high levels of sucrose storage occurs only in the culm parenchyma cells as against in other plants where storage of sugar or other storage molecule/s occurs in terminal sink organs such as tubers, grains, or fleshy fruits. Sucrose concentration that peaks in the sugarcane culm during the end of the vegetative cycle (called ripening) is utilized for the sexual reproductive phase and the remaining reserve is re-mobilized to produce new vegetative structures unlike the pattern in monocarpic annuals where there is a single cycle of storage and utilization for the reproductive phase [2]. In addition, sucrose is the only major form in which reduced carbon is exported from the source and hence all cellular processes outside the source are dependent on the mobilisation and utilisation of sucrose. Sucrose is the dominant storage reserve in sugarcane in contrast to most other plant stems that store polysaccharides such as starch or fructans with a low concentration of sucrose. As sugarcane matures, there is a shift in carbon partitioning from that of insoluble and respiratory components towards the osmotically active sucrose [3].

Although sugarcane stores the highest concentration (reaching about 0.7 M) of sucrose in the plant kingdom, studies on the physiological, biochemical and genetic basis of sucrose synthesis and accumulation have been limited compared to those in model plants like Arabidopsis or rice that do not accumulate high levels of sucrose. There are very few studies of sucrose accumulation primarily focusing on the sugarcane culm. Often these studies in sugarcane have reported a network of genes related to cell wall metabolism, carbohydrate metabolism, stress responses and regulatory processes [4–11]. Microarray analysis of sugarcane genotypes that varied in sucrose content revealed that many of the genes associated with high sucrose content showed overlap with drought data sets, but appeared to be mostly independent from abscisic acid signalling [12]. A large expressed sequence tag (EST) study of the sugarcane transcriptome and physiological, developmental and tissue-specific gene regulation was initiated in Brazil [13]. Sugarcane cultivars differing in both maximum sucrose accumulation (in Brix) capacity and accumulation dynamics during growth and culm maturation were studied cDNA microarrays and developmentally regulated genes related to hormone signalling, stress response, sugar transport, lignin biosynthesis and fibre content were identified [12]. An expression profiling of a set of genes associated with sucrose accumulation was studied using quantitative real time reverse transcription PCR (qRT-PCR) in 13 genotypes of sugarcane and its progenitor species including *S*. *officinarum*, *S. spontaneum* and related genera *Erianthus arundinaceus* [14]. High brix genotypes exhibited increased expression of sucrose non-fermenting related kinases and cellulose synthases in an expression study comparing high and low brix genotypes of sugarcane using qRT-PCR [15]. In another transcriptome study using next generation sequencing (NGS) [16] enrichment of transcripts involved in a network of sucrose synthesis, accumulation, storage and retention in relation to the agronomic characteristics of the genotypes contrasting for rust resistance was observed. Casu et al. [9] proposed that sucrose accumulation may be regulated by a network of genes induced during culm maturation which included clusters of genes with roles that contribute to key physiological processes including sugar translocation and transport, fibre synthesis, membrane transport, vacuole development and function, and abiotic stress tolerance. These studies show that the sugarcane culm is a composite organ associated with numerous diverse functions other than sucrose storage. A gene networking pattern involving genes associated with culm maturation and sucrose accumulation, sugar transport, vacuole development, lignification, suberisation and abiotic-stress tolerance can be inferred from these studies. The present study aimed to identifying transcripts that were associated with sucrose accumulation using a set of seven high sugar and seven low sugar genotypes by expression profiling of mature and immature culm tissues and bioinformatic analyses of culm transcriptomes. The upregulation of several thousands of transcripts associated with sucrose biosynthesis was demonstrated in the high sugar, and maturing culm of sugarcane. This is the first transcriptome study showing the association of expression of a large number of genes with sucrose synthesis and accumulation in the sugarcane culm tissue.

## Methods

### Plant material and phenotypic data collection

Fully grown, disease free 12 months old plants grown in the field in a randomized complete block design were selected for analysis. The genotypes were derived from a sugarcane population provided by Sugar Research Australia (SRA), Brisbane, Australia, previously described in [17]. Sugar content measured as Brix (a measure of the soluble solids in sugarcane juice) was used for classifying the genotypes as high and low sugar genotypes (Table 1). The low sugar genotypes had a Brix range of 17–18.4 while the high sugar genotypes had a Brix range of 19.4–21.4. The Brix at the point of collection was used for defining the high and low sugar groupings. These genotypes may have high or low sugar content in other environments. A wide variation in sugar content was not obtained as these genotypes were commercial cultivars and introgression lines in the breeding pipeline with a sugar content above 16 and fibre content below 15 on a fresh weight basis. Culm samples (from both top and bottom tissues, the 4th internode from top and 3rd internode from the bottom of the cane) were collected from four representative stalks and pooled for each internode sample. All samples were collected between 10 am to 2 pm to limit the diurnal fluctuations in the transcriptome. After collection, the samples were immediately flash frozen in liquid nitrogen and stored at −80 °C until RNA extraction. In addition, HPLC (high performance liquid chromatography) and NIR (near infrared spectroscopy) was used to measure the sugar composition and fibre content on a fresh weight basis. A sub-sample of each genotype was processed through a mechanical grinder, a component of the SpectraCane system (Biolab, Australia) and scanned by NIR for fibre content, Brix and sugar content (commercial cane sugar - CCS). For details see Additional file 1: Tables S1a, S2, S3; Figure S1.

**Table 1:** Sugar, Brix, fibre and pedigree information for each genotype

Genotype	Code	Brix (degree)	Fibre (%)	Pedigree	Group
QC02–402	G01	18.3	31.39	Commercial hybrid	Low sugar
QA02–1009	G02	18.3	43.36	Commercial hybrid	Low sugar
QN05–803	G10	17.8	47.74	Commercial hybrid	Low sugar
KQB07–24739	G16	18.4	48.2	Introg. BC1 (*S. spont*)	Low sugar
KQB09–23137	G18	17.7	33.53	BC1 (*S. spont*)	Low sugar
KQB09–20620	G19	17.8	39.88	Introg. BC1 (*S. spont*)	Low sugar
KQB09–20432	G20	18.3	49.79	Introg. BC1 (*S. spont*)	Low sugar
QN05–1743	G04	21.4	34.62	Commercial hybrid	High sugar
QN05–1509	G05	20.1	40.43	Commercial hybrid	High sugar
QS99–2014	G06	20.9	31.21	Commercial hybrid	High sugar
QA96–1749	G07	19.4	46.33	Commercial hybrid	High sugar
Q200	G09	19.7	37.6	Commercial hybrid	High sugar
KQB07–23990	G13	20	36.25	Introg. BC1 (*S. spont*)	High sugar
KQ08–2850	G14	20.3	43.84	Introg. BC3 (*Erianthus* sp)	High sugar

Introg-introgression; BC-back cross; *S. spont*- *Saccharum spontaneum*

### Sample collection and preparation for RNA-Seq

The frozen sugarcane samples were pulverized using a Retsch TissueLyser (Retsch, Haan, Germany) at a frequency of 30/S for 1 min 30 s and about 1 g of ground sample powder was used for RNA extraction. RNA extractions were conducted as described by Furtado et al. (2014) [18] employing a Trizol kit (Invitrogen) and a Qiagen RNeasy Plant minikit (#74134, Qiagen, Valencia, CA, USA). For RNA quality and quantity assessment, a NanoDrop8000 spectrophotometer (ThermoFisher Scientific, Wilmington, DE, USA), and an Agilent Bioanalyser 2100 with the Agilent RNA 6000 Nano kit (Agilent Technologies, Santa Clara, CA, USA) were used. Only RNA samples with a RIN value of >7.5 were chosen for library preparations. About 3 μg each of 28 internodal RNA samples was used for indexed-library preparation (average insert size of 200 bp) with a TruSeq stranded with Ribo-Zero Plant Library Prep Kit for preparing total RNA library (Illumina Inc.) as described in [19]. The library was subjected to sequencing in two lanes (equimolar) using an Illumina HiSeq4000 instrument to obtain paired-end (PE) read of 150 bp. The library preparation and sequencing was conducted at the Translational Research Institute, The University of Queensland, Australia.

### RNA-Seq data processing

Read adapter and quality trimming were performed in CLC Genomics Workbench v9.0 (CLC-GWB, CLC Bio-Qiagen, Aarhus, Denmark) with a quality score limit of <0.01 (equivalent to Phred Q score ≥ 20), and allowing a maximum of two ambiguous nucleotides. Only PE reads with a length ≥ 35 bp were kept for further analyses. Further information on the RNA Bioanalyser profiles, raw RNA-Seq reads, trimming, quality parameters including size distribution and GC content is described in detail in [19] and in Additional file 1; Table S1b. Table 2 gives the details of reads from each genotype (top and bottom internode tissues) after quality trimming.

**Table 2:** RNA sequence data obtained for each genotype

Low sugar genotypes			High sugar genotypes		
Code	Number of paired end reads		Code	Number of paired end reads	
	Top	Bottom		Top	Bottom
G01	19,224,245	41,184,328	G04	24,837,412	22,869,941
G02	42,714,286	79,760,012	G05	8,578,357	47,973,820
G10	15,278,340	80,811,427	G06	12,971,379	24,967,893
G16	28,875,842	46,037,690	G07	25,204,740	23,694,629
G18	64,789,858	66,628,598	G09	36,810,635	36,366,607
G19	24,882,334	30,954,783	G13	19,443,531	24,196,385
G20	6,529,582	45,500,054	G14	37,845,062	19,172,922

### Differential gene expression (DGE) analyses

Using the CLC-GWB v9.5.1 software, RNA-Seq experiments were performed with a minimum length fraction of 0.9 and a minimum similarity fraction of 0.8. The number of reads per kilobase per million mapped reads (RPKM) was used for normalization [20]. The CLC-GWB provides a comprehensive RNA-Seq tool for differential gene expression accompanied by statistical analyses. The Baggerley’s test that is used in this case [21] is the proportion-based statistical analysis that uses raw count data (un-transformed, not-previously-normalized) as input for setting up the experiment and uses total or unique gene/exon reads for calculating the differentially expressed genes. This test compares counts by considering the proportions that the counts for each gene make-up of the total sum of counts in each group. That is, it takes into account the proportion of every genotype in a group for a gene to be considered as differentially expressed. When Edge test [22] in CLC-GWB (an equivalent tool to EdgeR available in R Package v3.4.0) was used, this consistency (of differential expression across all genotypes in a group) was not observed. Similarly, the Differential expression for RNA-Seq tool available in the recent version of CLC GWB 10.1.1 gave a different set of results for the DGE experiments (data not shown here) and hence was not included for further analyses. As sugarcane genotypes differ genetically among and between each other, the criterion was to select only those genes that were differentially expressed despite the genetic differences inherent to the genotypes. For example, a gene was considered differentially expressed only when it is consistently differentially expressed in all the seven genotypes in one group in comparison with all the seven genotypes in the other group. Further the Baggerley test also corrects for the differences in the sample sizes (within and between library variations) by comparing the expression levels at the level of proportions rather than raw counts [21]; CLC manual).

**Fig. 1:**
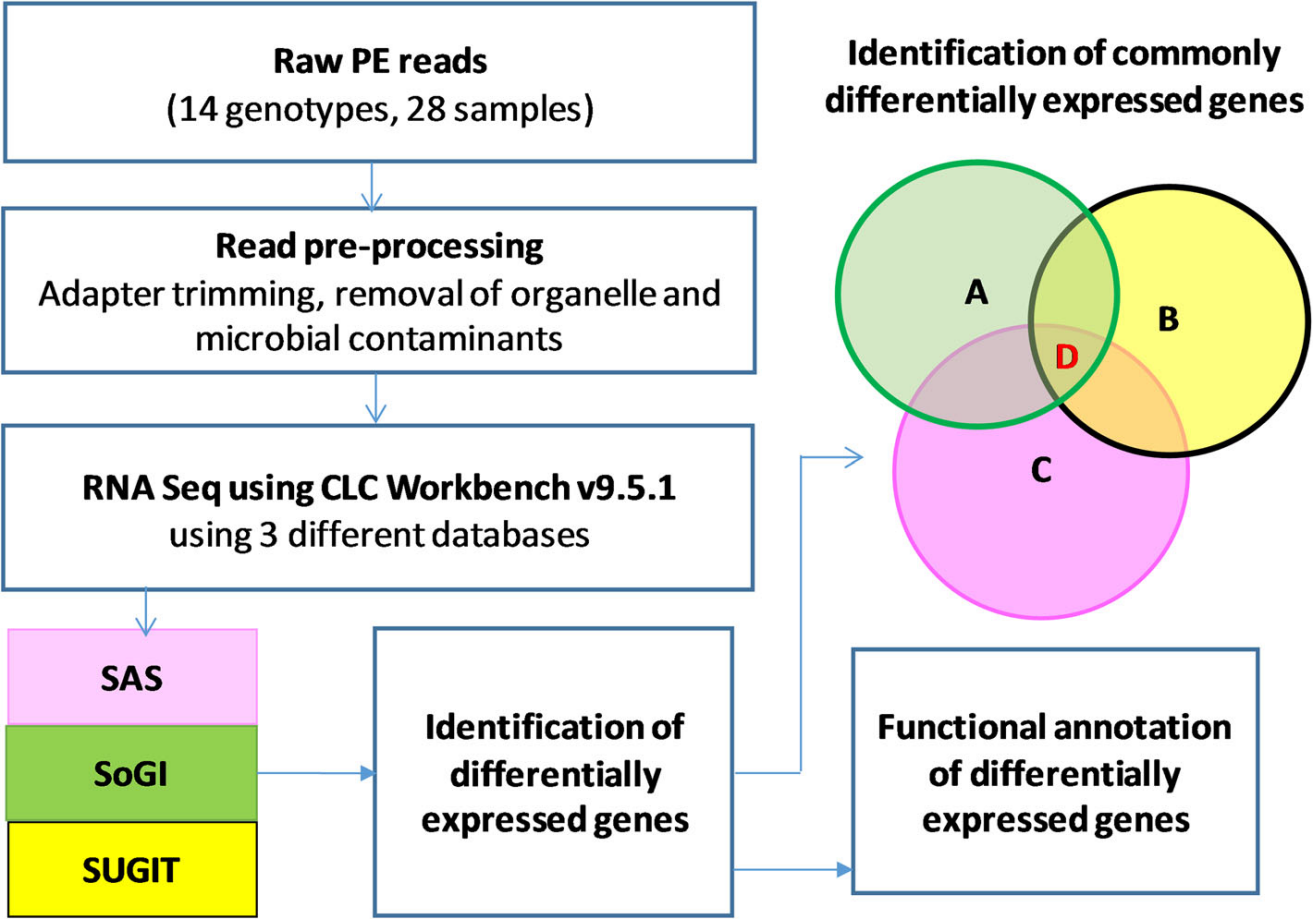
Workflow for transcriptome sequencing, RNA-Seq experiments and the identification of differentially expressed transcripts. High and low sugar genotypes as well as mature and immature culm samples of sugarcane genotypes were compared in the study

The reads for each genotype in the high and low sugar groups were separately mapped against reference databases, the *Saccharum officinarum* gene indices (SoGI), the sugarcane Iso-Seq transcriptome database (SUGIT, TSA accession number GFHJ01000000) and the sugarcane assembled sequences (SAS) from the sugarcane expressed sequence tags database (SUCEST). The SoGI database was downloaded from the DFCI gene indices [23] which had adequate gene or protein function descriptions. In the present case, the SoGI dataset represented 282,683 ESTs that resulted in 121,342 unique sequences after clustering. A collection of ∼240,000 ESTs generated by the SUCEST project from 26 cDNA libraries from different sugarcane tissues sampled at various developmental stages [24] were assembled into 43,141 distinct contigs using CAP3 [25]. This set of 43,141 contigs make up the SAS database. The SAS database was not annotated and the annotation was performed using the BLASTX against the nr protein database with an e value of 10–^5^, for 100 hits using the high-performance computing facility (HPC), at The University of Queensland, Australia. In addition, we used a newly constructed SUGIT, sugarcane long reads database described in [26]. In brief, the database was derived from a pooled RNA sample collection including those genotypes used in this study, plus leaf and root tissue samples of 22 commercial and introgressed sugarcane genotypes. The basic descriptions of the databases are given in Table 3 and the methodology is summarised in Fig. 1.

**Table 3:** Databases used for RNA Seq experiments

Features	SoGI	SUGIT	SAS
Total number of contigs	121,342	107,598	43,141
Total number of bases	88,397,709	166,929,028	35,730,322
Longest contig (bp)	4854	18,858	6193
Shortest contig (bp)	100	307	56
N50 (bp)	729	1994	827
N75 (bp)	642	1269	641

SoGI-*Saccharum officinarum* gene indices; SUGIT-Sugarcane Iso-Seq transcriptome database; SAS-sugarcane assembled sequences; bp- base pairs

### Identification of differentially expressed transcripts

For all the RNA-Seq experiments, involving high and low sugar groups, low sugar samples were used as the references, for comparing top and bottom (immature and mature culm tissues respectively), bottom internode sample was used as the reference for identifying DEGs that were upregulated or down regulated. This means, if one transcript was up-regulated in the reference group, it was down-regulated in the group being compared, and vice versa. Proportion based statistical analysis (Baggerley’s Test) and a Volcano plot were used to compare gene expression levels in the two groups that were considered for differential gene expression (high and low sugar, top and bottom internode samples) in terms of the log_2_ fold change (at FDR 0.01). The DEGs were further sorted and selected at three different fold change levels, i.e., above and equal to 2, above and equal to 10 fold and below 2 fold change to identify highly expressed and those expressed at low levels.

### Functional annotation of identified differentially expressed transcripts

Functional annotation of the transcripts was performed using MapMan categories [27] using BlastX (e-value ≤10^−5^, with a cut off value of 80% similarity) against *Arabidopsis thaliana* and *Oryza* sp. and SwissProt/UniProt Plant Proteins. In addition Blast2GO [28] followed by KEGG pathway mapping analyses were performed for the DEGs.

### Validation of gene expression using quantitative real-time PCR (qPCR)

In addition, a correlation analysis was performed to validate the expression levels of eight selected transcripts from the RNA-Seq analyses in this study using qPCR expression values of the same transcripts extracted from a separate study [29]. The RPKM values obtained for four tissue samples (two top and two bottom internodes) of two genotypes (QC02–402 and QN05–803) were correlated against the respective qPCR expression values (Cq qPCR normalised gene expression), using Microsoft Excel 2013.

## Results

### RNA-Seq analyses and identification of differentially expressed genes

**Fig. 2:**
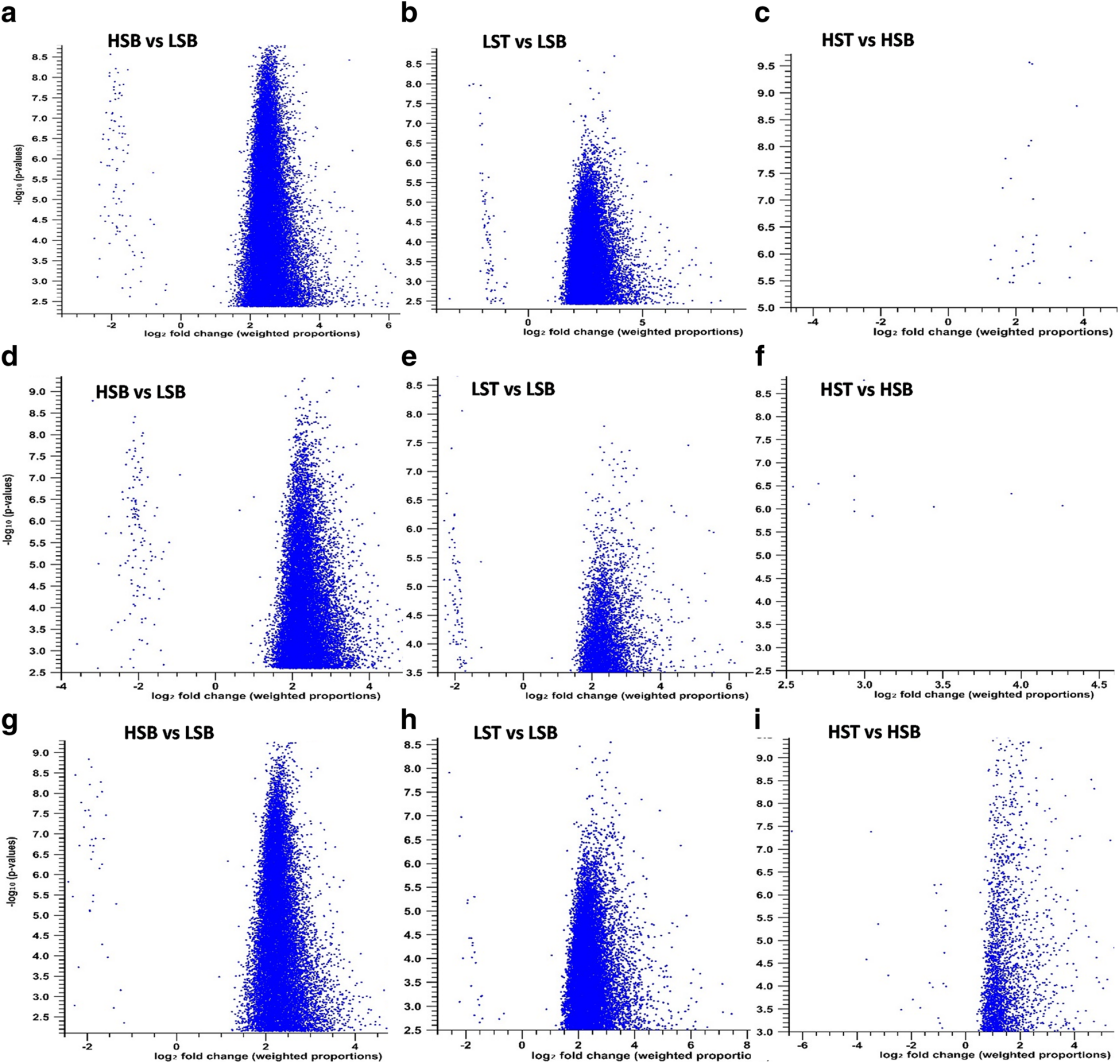
Volcano plot depiction of the DGEs in different groups using **a, b, c)** *Saccharum officinarum* gene indices, SoGI; **d, e, f)** Sugarcane Iso-Seq transcriptome database, SUGIT; **g, h, i)** Sugarcane assembled sequences, SAS. LST, low sugar top internode; LSB, low sugar bottom internode; HST, high sugar top internode; HSB, high sugar bottom internode; In HSB vs LSB, HSB shows upregulation of transcripts, whereas in LST vs LSB, LST shows upregulation of transcripts; in HST vs HSB, HSB shows a clear upregulation of transcripts using SAS database, though very few transcripts showed differential expression in other two databases. Please note that there was no DGE detected in HST vs LST

**Fig. 3:**
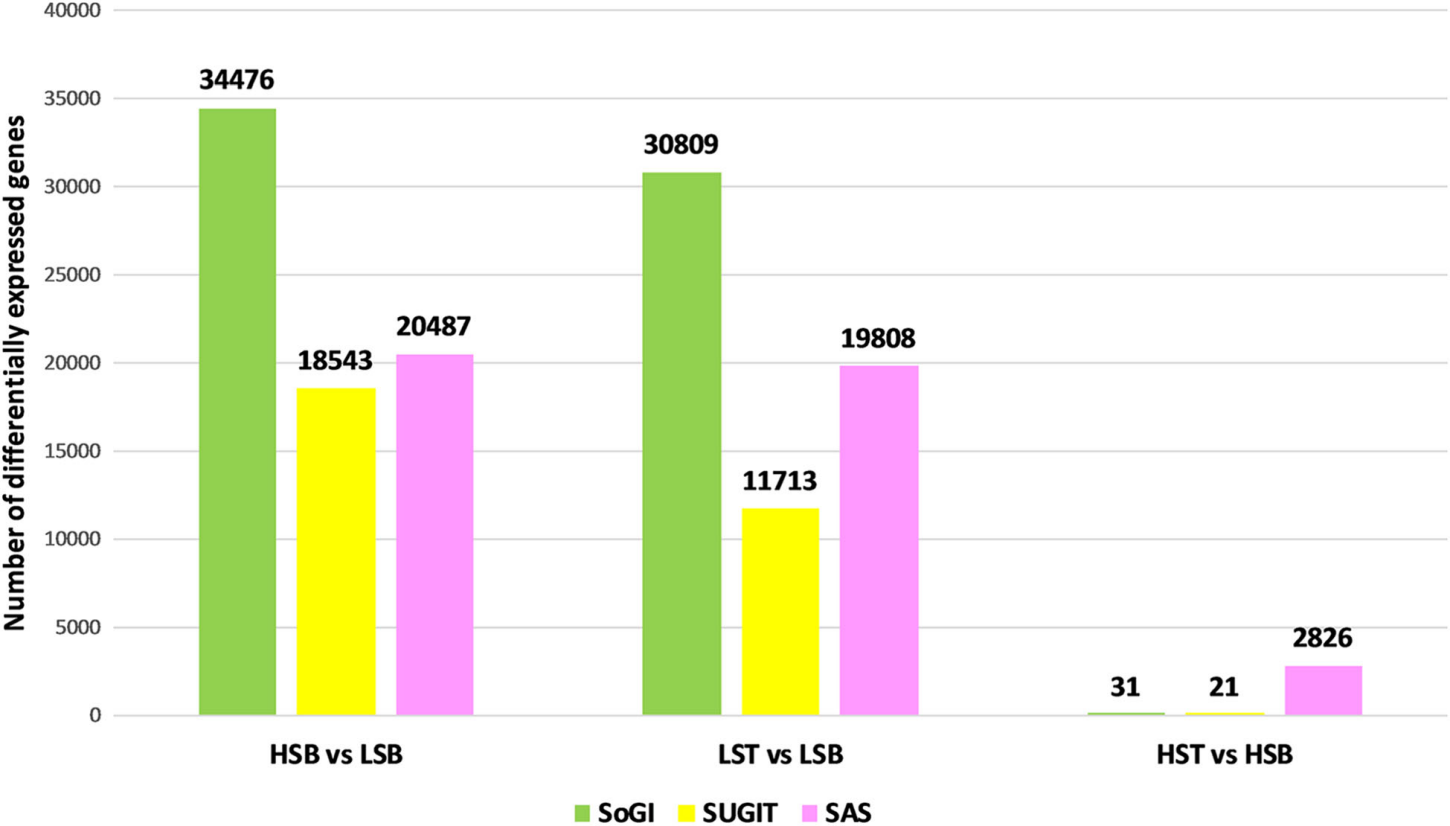
Graphical representation of DGE experiments with three different databases, *Saccharum officinarum* gene indices, SoGI; Sugarcane Iso-Seq transcriptome database, SUGIT; Sugarcane assembled sequences, SAS. LST, low sugar top internode; LSB, low sugar bottom internode; HST, high sugar top internode; HSB, high sugar bottom internode

**Fig. 4:**
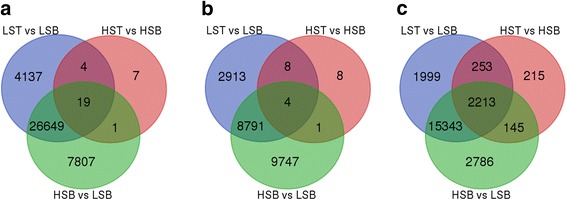
Venn diagrams depicting the common and unique DEGs obtained from three different comparisons between mature and immature culm tissues of high and low sugar genotypes. LST, low sugar top internode; LSB, low sugar bottom internode; HST, high sugar top internode; HSB, high sugar bottom internode; **a**, **b** and **c)**, RNA-Seq using *Saccharum officinarum* gene indices, SoGI; Sugarcane Iso-Seq transcriptome database, SUGIT; Sugarcane assembled sequences, SAS, respectively

**Fig. 5:**
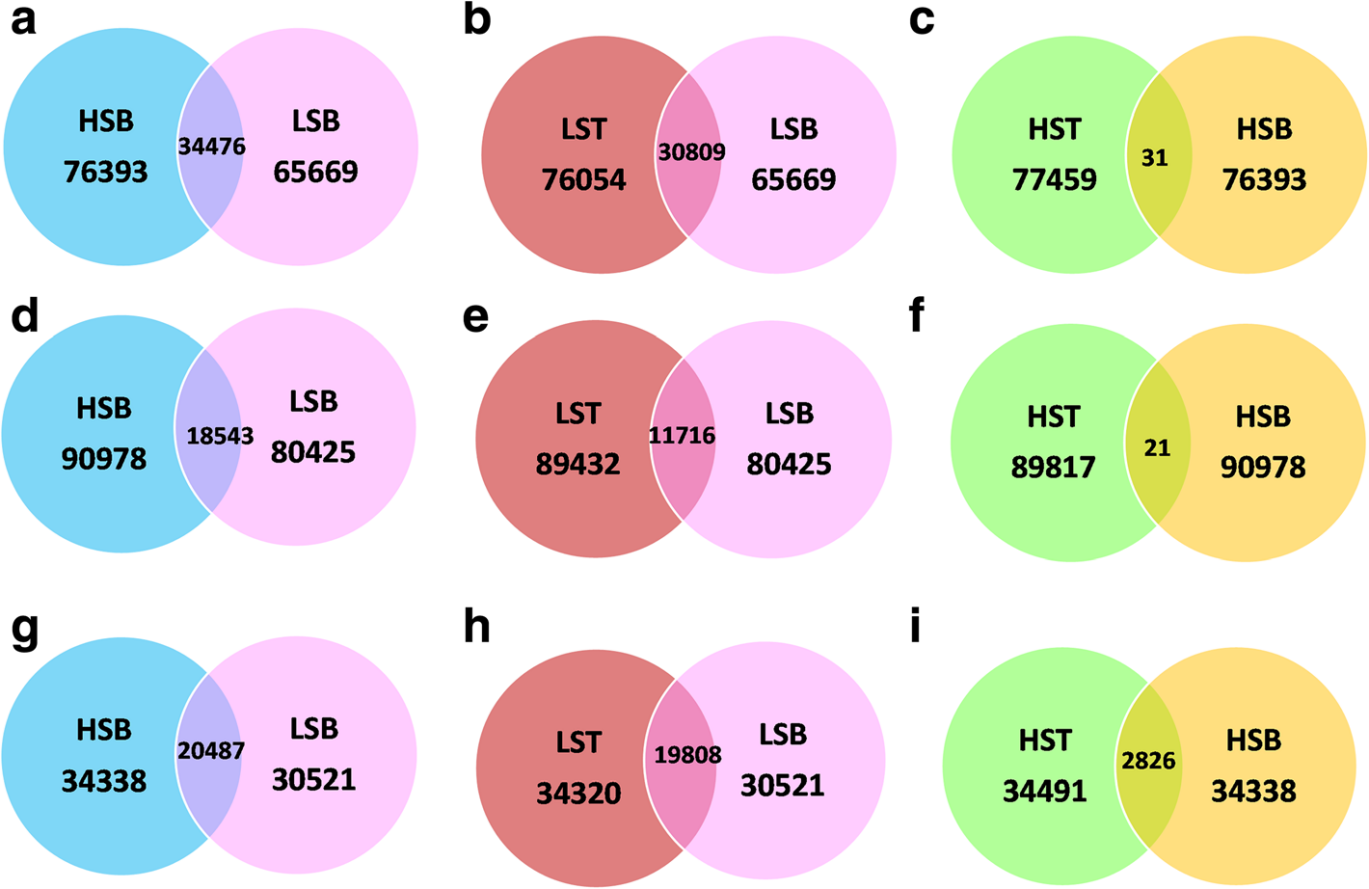
A schematic representation of global and differential gene expression between and within the groups using three databases. **a, b, c**) *Saccharum officinarum* gene indices, SoGI (121,342 ESTs); **d, e, f**) SUGIT-Sugarcane Iso-Seq transcriptome database (107,598 contigs) and **f, g, h**) Sugarcane assembled sequences, SAS (43,141) contigs**.** LST, low sugar top internode; LSB, low sugar bottom internode; HST, high sugar top internode; HSB, high sugar bottom internode. The numbers within the intersection are the number of DEGs between the groups compared. The numbers in the circle gives the number of transcripts expressed against the reference database with a RPKM cut off of >0

The mapping of reads to each reference database is shown in the Table 4. The results of the different RNA-Seq experiments (hereafter SoGI-DGE, SUGIT-DGE and SAS-DGE) are listed in Table 5 and the differential gene expression patterns are depicted as Volcano plots in the Fig. 2. For all DEGs, FDR 0.01 and a fold change of ≥2 were used as cut off values. In SoGI-DGE, with high and low sugar bottom internode samples (HSB vs LSB), out of the total 121,342 transcripts, 34,375 showed upregulation and 101 transcripts showed down regulation in high sugar genotypes when compared to low sugar genotypes. When low sugar top and low sugar bottom internode samples (LST vs LSB) were compared, 30,723 transcripts were differentially expressed, upregulated in the low sugar top internode sample, while 86 transcripts were down regulated. When high sugar top and high sugar bottom intermodal samples (HST vs HSB) were considered, 31 transcripts were found to be upregulated in high sugar bottom internode sample compared to the corresponding top. In SUGIT-DGE, out of 107,598 transcripts, 18,411 transcripts were upregulated while 132 transcripts were down regulated in high sugar bottom internode sample compared to low sugar bottom internode sample (HSB vs LSB). 11,713 transcripts were differentially expressed between low sugar top and low sugar bottom intermodal samples (LST vs LSB), wherein 11,599 transcripts were upregulated and 114 transcripts were down regulated. In the SAS-DGE, 19,808 transcripts showed differential expression (19,782 upregulated, 26 down regulated) out of 43,141 transcripts of the SAS reference database in the HSB vs LSB comparison and 20,487 transcripts were differentially expressed (20,449 up regulated, 38 down regulated) in the LST vs LSB comparison (see Table 5 for details). However, in the SAS-DGE, there were more DEGs in the HST vs HSB comparison, with 2826 DEGs. This comparison resulted in only 21 and 31 DEGs with the SUGIT and SoGI DGEs respectively (Fig. 3). In addition, the common and unique transcripts among the three comparisons in three different DGEs were found (Figs. 4 and 5). For additional information on experimental set up and statistical analyses, see Additional files 2: Table S4-S6 (complete list of DEGs in the SOGI-DGE), Additional files 3: Table S7-S9 (complete list of DEGs in the SUGIT-DGE), and Additional files 4: Table S10-S12 (complete list of DEGs in the SAS-DGE). The results of the qPCR expression values were found to be significantly correlated with the RPKM values for selected genes (*r* = 0.629, *p* < 0.001, *n* = 32, df = 30). The details of qPCR validation analysis are provided in Additional file 5: Table S13 and Figure S2.

**Table 4:** Reads from each genotype with details of mapping to three different databases SoGI, SUGIT and SAS

Code	Top internode				Bottom internode			
	Trimmed reads	Reads mapped (%)			Trimmed reads	Reads mapped (%)		
		SoGI	SUGIT	SAS		SoGI	SUGIT	SAS
High sugar genotypes								
G04	24,837,412	52.12	70.65	54.86	22,869,941	46.03	69.38	52.64
G05	8,578,357	48.63	69.34	57.65	47,973,820	49.63	69.35	52.56
G06	12,971,379	52.41	74.20	56.09	24,967,893	47.72	71.51	54.77
G07	25,204,740	51.85	70.79	55.45	23,694,629	50.42	70.45	55.07
G09	36,810,635	52.11	73.64	56.87	36,366,607	54.16	80.44	56.56
G13	19,443,531	52.45	69.10	51.22	24,196,385	50.35	68.45	54.57
G14	37,845,062	52.38	67.85	56.98	19,172,922	51.38	68.90	55.90
Low sugar genotypes								
G01	19,224,245	47.95	70.76	54.87	41,184,328	52.45	78.06	55.75
G02	42,714,286	45.45	66.43	52.42	79,760,012	54.89	70.48	57.26
G10	15,278,340	51.09	72.10	54.05	80,811,427	55.42	81.09	56.65
G16	28,875,842	52.19	75.47	56.07	46,037,690	55.19	80.31	56.07
G18	64,789,858	54.13	71.65	56.69	66,628,598	54.47	72.14	53.71
G19	24,882,334	46.73	67.67	54.39	30,954,783	56.50	72.57	56.97
G20	6,529,582	55.51	73.44	55.82	45,500,054	55.51	80.15	55.82

SoGI-*Saccharum officinarum* gene indices; SUGIT-Sugarcane Iso-Seq transcriptome database; SAS-sugarcane assembled sequences

**Table 5:** Details of differentially expressed genes obtained from three different RNA-Seq experiments at FDR 0.05 and 0.01

Experiment	FDR 0.01			FDR 0.05			
	Up	Down	Total	Up	Down	Total	Reference
SoGI-DGE							
HST vs HSB	–	31	31	1	58	59	HSB
HSB vs LSB	101	34,375	34,476	111	43,109	43,220	LSB
LST vs LSB	86	30,723	30,809	102	42,149	42,251	LSB
SUGIT-DGE							
HST vs HSB	–	21	21	–	38	38	HSB
HSB vs LSB	132	18,411	18,543	140	30,129	30,269	LSB
LST vs LSB	114	11,599	11,713	142	26,626	26,768	LSB
SAS-DGE							
HST vs HSB	2591	235	2826	4752	383	5135	HSB
HSB vs LSB	38	20,449	20,487	41	24,233	24,274	LSB
LST vs LSB	26	19,782	19,808	36	24,438	24,474	LSB

HSB, high sugar bottom internode; HST, high sugar top internode; LSB-low sugar bottom internode; LST-low sugar top internode; SoGI-*Saccharum officinarum* gene indices; SUGIT-Sugarcane Iso-Seq transcriptome database; SAS-sugarcane assembled sequences

### Identification of consistently differentially expressed transcripts between high and low sugar genotypes

The results of the DGE analyses are given in the Table 5. The DEGs at different fold change cut off values, i.e. ≥2, ≥10 and <2 fold changes were identified. This resulted in the identification of DEGs that are expressed at high levels (10 fold and above), low levels (<2) apart from the cut off of 2 and above (Table 6). In addition, to check for specific sucrose/sugar related transcripts, filtering was done for “sucrose” and “sugar” as key words in the DGE experiment files as the DEGs were in large numbers. Although some transcripts related to sucrose/sugar may have been missed, this approach helped screening the large number of DEGs. At the fold change value of 2 and above, the sucrose and sugar related genes were 63, 68 and 49 in HSB vs LSB and 75, 74, and 60 in in LST vs LSB using SoGI-DGE, SUGIT-DGE and SAS-DGE respectively. These transcripts are listed in the Additional file 6: Tables S14–22 and some are listed in the Tables 7, 8 and 9. At the fold change value of 10 and above, the sucrose/sugar related transcripts were very few in number and included sucrose synthase (SuSy), sucrose transporter (SuT), sucrose phosphate synthase (SPS) and a SWEET transporter (Table 6). Further, SuSy2 and SuT3 were consistently present in all three sets of DEGs for LST vs LSB, at the maximum fold change value of 10 and above, showing upregulation in LST. In HSB vs LSB, SuSy 2 and SuT 2 were observed in SoGI-DGE and SUGIT-DGE, upregulated in HSB, whereas no sucrose/sugar related transcripts were present in SAS-DGE at this fold change. At the fold change value of below 2, there were no sucrose/sugar related transcripts for these two comparisons in any of the DGEs. In HST vs HSB, sucrose/sugar related transcripts were not found in SoGI- and SUGIT-DGEs, however, at the fold change cut off value of below <2, sucrose phosphate phosphatase (SPP) 2, SuSy, SWEET 16 like transporter and a sugar phosphate phosphate translocator were found in SAS-DGE, showing upregulation in HSB. Interestingly, the DEGs at fold change ≥10 in HST vs HSB were related to phenyl propanoid pathway genes like terpene cyclase (TC), phenyl ammonia lyase (PAL), chalcone synthase (CHS), cinnamoyl CoA reductase (CCoAR), ferruloyl esterase (FE), laccase 7-like (LAC), β-expansin (BE) 1a and ethylene responsive transcripts etc., in SoGI, SUGIT and SAS-DGEs (Table 6). Overall, genes specific to sucrose synthesis and accumulation were enriched in the HSB vs LSB and LST vs LSB experiments, while genes for secondary metabolites, were found to be enriched in the case of the HST vs HSB comparison. There were no DEGs in HST vs LST experiment in the three DGE analyses.

**Table 6:** Details of differentially expressed genes obtained from three different RNA seq experiments at FDR 0.01 at three different fold change settings

	HSB vs LSB			LST vs LSB			HST vs HSB		
	Fold change			Fold change			Fold change		
	≥2	≥10	<2	≥2	≥10	<2	≥2	≥10	<2
SoGI- DGE	(34476) *SuSy 2* ***SuT2***	(1723) ***SuSy 2*** ***SuT2***	(7)	(30809) ***SuSy 2*** ***SuT2***	(3279) ***SuSy 2*** ***SuT***	(5)	(31)	(5) *PAL* *CHS* *CCoAR*	–
SUGIT- DGE	(18543) ***SuSy 2*** ***SuT2a***	(952) ***SuSy 2*** ***SuT2a***	(2)	(11716) ***SuSy 2*** ***SuT2***	(872) ***SuSy 2*** ***SuT3***	(2)	(21)	(3) *TC*	–
SAS-DGE	(20487) ***SuSy 2*** ***SuT3***	(575)	(4)	(19808) ***SuSy 2*** ***SuT2***	(1706) ***SuSy 1*** ***SuT3*** *SWEET3* *SPS1*	(2)	(2826)	(74+) (3-) *FE, LAC, BE*	(794+) (172-) *SPP2* *SWEET3 SWEET16* *SPT*

*SuSy*-sucrose synthase; *SuT*-sucrose transporter; *SWEET*-bidirectional sugar transporter sweet; *FL*-Ferruloyl esterase; *LAC*-laccase; *BE*-beta expansin; *SPP*-sucrose phosphate phosphatase; *SPT*-sugar phosphate phosphate translocator; HSB, high sugar bottom internode; HST, high sugar top internode; LSB-low sugar bottom internode; LST-low sugar top internode; SoGI-*Saccharum officinarum* gene indices; SUGIT-Sugarcane Iso-Seq transcriptome database; SAS-sugarcane assembled sequences. The numbers in brackets indicate the number of DEGs obtained at that fold change setting, while the sucrose and sugar related genes within the DEGs are indicated below them. (+) and (−) denote upregulation and down regulation respectively. The genes in bold letters are present in all the three DEGs

**Table 7:** DEGs obtained between high sugar bottom (HSB) vs low sugar bottom (LSB) internode samples using three databases SoGI, SUGIT and SAS. Shown here are some of the sucrose/sugar related transcripts

Feature ID	Description	Fold change (original values)	**FDR < 0.01**
SoGI-DGE			
CA255667	Sucrose synthase 2	−28.85	0.01
CA207180	Sucrose transporter 2	−18.14	5.53E-03
CA267680	Sugar-phosphate isomerase-like protein	−17.31	4.84E-03
TC112923	Sugar-starvation induced protein	−15.38	7.76E-03
TC121981	Sucrose synthase 3	−8.42	1.88E-04
TC146639	UDP-sugar pyrophosphorylase	−8.39	3.65E-04
TC113610	Sucrose phosphate phosphatase	−8.29	1.66E-03
CA072415	Sucrose non-fermenting related protein kinase	−7.5	3.33E-05
CA258700	Possible sugar transferase	−6.69	1.04E-04
TC140141	Impaired sucrose induction 1-like protein	−6.53	4.84E-04
CA233504	Sugar phosphate exchanger 2	−6.15	1.68E-03
TC131675	Sucrose phosphate synthase III	−5.74	2.64E-06
TC136732	Sucrose transporter SUT4	−5.49	8.38E-06
TC146044	Sugar transporter ERD6-like 5	−5.21	3.08E-06
TC129039	Sugar efflux transporter	−4.92	1.24E-04
SUGIT-DGE			
c94324f1p42760	sugar transporter type 2a	−12.62	9.96E-03
c98328f1p02743	Sucrose synthase 2	−10.46	3.32E-03
c98146f1p0774	sugar transporter (ERD6)	−9.43	1.00E-02
c88771f1p01741	Bidirectional sugar transporter SWEET	−9.2	3.59E-03
c32435f3p21876	Sucrose non-fermenting related kinase 1b	−8.47	8.64E-03
c41415f1p01118	Sucrose transporter 1	−6.16	6.80E-03
c29857f1p01086	UDP-sugar pyrophosphorylase	−5.72	2.93E-04
c106308f1p04384	Sucrose phosphate synthase A	−5.33	2.54E-04
SAS-DGE			
SCUTFL1058E04.g	sugar phosphatase -like	−7.8	6.02E-04
SCEQAM1036A06.g	sucrose-phosphate synthase 3	−7.47	4.37E-06
SCEZAM2031D12.g	UDP-sugar pyrophosphorylase	−7.29	2.74E-04
SCEQRT1031C11.g	bidirectional sugar transporter SWEET14-like	−7.17	2.06E-03
SCEPAM2014B12.g	sucrose transport SUC3	−7.08	2.03E-05
SCEPCL6023F02.g	sucrose synthase 2	−6.89	2.30E-04
SCBGSD2049G08.g	sugar transport 7	−6.72	8.51E-05
SCAGLR1021A01.g	sugar phosphate phosphate translocator	−6.34	9.12E-05
SCCCRT2001F10.g	sucrose non-fermenting 4	−6.08	1.81E-06
SCCCLR1C06G07.g	sucrose-phosphate synthase 1	−5.63	4.97E-06
SCCCRZ1004G04.g	impaired sucrose induction 1	−5.21	7.47E-05
SCEPLR1008A12.g	sucrose transport SUC4-like	−4.81	4.82E-06
SCSBST3096E12.g	sucrose-phosphatase 2-like	−5.21	3.55E-05

SoGI-*Saccharum officinarum* gene indices; SUGIT-Sugarcane Iso-Seq transcriptome database; SAS-sugarcane assembled sequences

**Table 8:** DGEs obtained between low sugar top (LST) and low sugar bottom (LSB) internode samples with three databases SoGI, SUGIT and SAS. Shown here are some of the sucrose/sugar related transcripts

Feature ID	Description	Fold change (original values)	FDR < 0.01
SoGI-DGE			
CA267680	Sugar-phosphate isomerase-like protein	−24.39	2.84E-03
TC123316	Sucrose synthase 2	−19.2	0.01
TC150523	Glycosyltransferase sugar-binding region	−11.03	0.01
TC153302	ADP-sugar diphosphatase	−8.41	7.81E-04
CA240368	Sucrose non-fermenting related protein kinase	−8.31	2.30E-03
TC141576	Sucrose phosphate phosphatase	−7.69	7.81E-04
CA136361	UDP-sugar pyrophosphorylase	−7.43	4.96E-03
TC140141	Impaired sucrose induction 1-like protein	−7.14	2.07E-03
TC136732	Sucrose transporter SUT4	−7.08	1.02E-03
TC113476	Sucrose phosphate synthase II	−6.44	9.73E-04
TC146044	Sugar transporter ERD6-like 5	−5.84	9.77E-04
CA233504	Sugar phosphate exchanger 2	−5.74	5.88E-03
TC117267	Sucrose phosphate synthase III	−5.61	9.44E-04
CA291037	Sucrose synthase 3	−5.4	1.86E-03
SUGIT-DGE			
c26397f1p01230	Sucrose synthase	−12.81	0.01
c96752f1p02674	sugar transporter type 2a	−9.5	0.01
c10824f1p0909	SUT2-h1	−7.52	0.01
c29857f1p01086	UDP-sugar pyrophosphorylase	−6.95	0.00131
c65976f2p01948	SUT4-h1	−6.6	0.0081
c1589f4p31134	Bidirectional sugar transporter SWEET	−5.14	0.00455
c42187f1p11882	Sucrose non-fermenting related kinase 1b	−5.44	0.01
c106308f1p04384	Sucrose phosphate synthase A	−5.12	0.00358
SAS-DGE			
SCJLHR1025D07.g	bidirectional sugar transporter SWEET3	−14.92	9.46E-03
SCCCRZ1002G07.g	sucrose-phosphate synthase 1	−13.59	9.84E-03
SCEQRT2090C11.g	Sucrose transport SUC3	−12.97	5.05E-04
SCQGST3153F06.g	sugar transport 5-like	−9.65	1.12E-03
SCCCLR2C03H09.g	sugar transporter ERD6-like 6	−9.36	1.34E-03
SCEZAM2031D12.g	UDP-sugar pyrophosphorylase	−9	3.23E-04
SCAGLR1021A01.g	sugar phosphate phosphate translocator	−8.78	3.46E-04
SCBGSD2049G08.g	sugar transport 7	−6.25	6.77E-03
SCCCRT2001F10.g	sucrose non-fermenting 4	−6.15	3.42E-04
SCEZRZ1013G04.g	Galactinol-sucrose galactosyltransferase 2	−6.09	1.07E-03
SCEPLR1008A12.g	sucrose transport SUC4-like	−5.94	2.87E-04
SCCCRZ1004G04.g	impaired sucrose induction 1	−5.76	5.49E-04
SCEZLR1031D07.g	sucrose-phosphatase 2	−5.59	2.62E-04
SCSGHR1068D07.g	UDP-sugar transporter	−4.91	5.61E-04
SCEPCL6023F02.g	sucrose synthase 2	−4.89	9.76E-04
SCEQAM1036A06.g	sucrose-phosphate synthase 3	−4.41	0.01

SoGI-*Saccharum officinarum* gene indices; SUGIT-Sugarcane Iso-Seq transcriptome database; SAS-sugarcane assembled sequences

**Table 9:** DEGs obtained between high sugar top (HST) vs high sugar bottom (HSB) internode samples with three different databases SoGI, SUGIT and SAS. Shown here are some of the sucrose/sugar related transcripts

Feature ID	Description	Fold change (original values)	FDR < 0.01
SoGI-DGE			
TC125737	Phenylalanine ammonia-lyase	−16.27	4.08E-03
TC131133	Chalcone synthase 5	−13.79	4.24E-05
CA207335	Cinnamoyl-CoA reductase	−12.11	5.19E-03
CA212197	Beta-amyrin synthase	−11.85	0.01
CA113829	LIM transcription factor homolog	−5.75	7.51E-03
CA065092	Universal stress protein family protein ERD65	−5.43	1.48E-09
TC124516	4-coumarate coenzyme A ligase	−4.59	4.16E-03
TC137240	Serine/threonine-protein kinase Nek5	−3.5	0.01
SUGIT-DGE			
c98442f1p02354	Terpene cyclase mutase family	−18.71	0.00608
c61441f1p11782	Phenylalanine ammonia lyase	−6.44	0.00382
SAS-DGE			
SCJFRT1010B12.g	Sugar phosphate phosphate translocator	2.43	3.49E-08
SCEZLR1031D07.g	Sucrose phosphate phosphatase 2	2.17	6.51E-03
SCACSB1117F03.g	Sucrose synthase	1.24	4.07E-03
SCEZSD1079C10.g	Bidirectional sugar transporter SWEET16-like	1.12	1.87E-04

SoGI-*Saccharum officinarum* gene indices; SUGIT-Sugarcane Iso-Seq transcriptome database; SAS-sugarcane assembled sequences

### Gene ontology annotation

The gene ontology annotation using MapMan resulted in grouping and classification of the DEGs into different functional categories. The DGE analysis between LST and LSB was almost similar in number and composition to the DEGs obtained between HSB vs LSB (Additional file 7: Figure S3, Table S23).

### Upregulated transcripts in high sugar genotypes when compared with low sugar genotypes

The DGE analyses of HSB vs LSB and LST vs LSB were showing a similar trend and the two sets of DEGs had an extensive overlap (see Additional file 7: Figure S3). About 89.3% (with SoGI) of the transcripts differentially expressed were similar in both the comparisons (63% in SUGIT and 96.8% in case of SAS). Hence only the DEGs of HSB vs LSB is considered for further discussion. Only a few transcripts are discussed here. For a complete list DEGs of all the three DGE analyses, refer Additional files 2, 3 and 4: Tables S4-S12. In addition, a list of unique and commonly expressed transcripts in each group was prepared (Additional File 8: Tables S24–28) for all the DGEs. The description below gives an overview of the DEGs obtained in the three DGE analyses at FDR 0.01 without any filtering.

#### Sucrose, starch and other sugar derivatives

In the SoGI-DGE, there were 71 sucrose related transcripts consisting of sucrose synthases 2 and 3, sucrose phosphate synthase (SPS) 2 and 3, sucrose phosphate phosphatase (SPP), *sucrose non-fermenting related* protein kinases; *impaired sucrose induction 1*-like protein and sucrose transporters (SuT) 2 and 4. About 22 transcripts were sugar related including transport, efflux, and glycosyltransferases. Ten transcripts were related to alkaline/neutral invertases and three transcripts with homology to sucrase from *Oryza sativa* were found. There were ten *high-glucose regulated protein 8-like* transcripts. Forty six transcripts, were related to intermediary metabolism of fructose phosphates, the most expressed being fructose-bisphosphate aldolase cytoplasmic isozyme. Sixteen transcripts were related to xylose metabolism and β-glucosidase related transcripts were observed. Fifteen hexose related transcripts were transporters, while 18 transcripts were related to triose phosphates metabolism. Fifty three UDP-related transcripts were found, out of which six were UDP-glucose-dehydrogenases. There were also UDP-sugar, arabinose, xylose, galactose transporters, −epimerases and -pyrophosphorylase related transcripts. Fucoses are hexose sugars and nine transcripts associated with them include fucosidases and fucosyltransferases. Thirteen mannose, trehalose and sorbitol related transcripts were found. Glucans metabolizing genes were another prominent group found to be highly expressed with 45 transcripts including β-1, 4 glucan synthases and endoglucanases. Nine transcripts related to alpha amylases were also upregulated in high sugar genotypes. In addition, 85 transcripts were found to be related to kinases including hexokinases, fructokinases (1, 2 and 3), phosphofructokinases, carbohydrate kinases and galactokinases. In the SUGIT-DGE, there were 208 sucrose related transcripts. In addition to the transcripts observed in the SoGI-DGE, sugar transport 5 and 7, sugar transporter ERD6 like, bidirectional sugar transporter SWEET1 and 4 like, and an abundance of ABC transporters B, C, D, E, G, F, and I for sugar were found in SUGIT-DGE. In the SAS-DGE, 75 transcripts were related to sucrose consisting of galactinol-sucrose galactosyltransferase 1,2 and 6, sucrose transporters SUC3 and its isoform X2, SUC4, SPP 2 and SPS 1, 3 and 4, bidirectional sugar transporter2a, 4, 14 and 16, sugar transporter ERD6-like 5, 6 and 16, sugar transport 5 and its isoform X1, 7 and 9 transcripts for sugar phosphate phosphate translocator. Interestingly one transcript for invertase inhibitor and one transcript for sulfofructose kinase like transcript which were not detected in the other two DGEs were found. Starch synthases II b and c, III, IV and starch branching and debranching (pullulanase and isoamylase) enzymes were found to be upregulated. The KEGG pathway map for starch and sucrose related DEGs are shown in the Additional file 9: Figure S4.

#### Vacuole and transporters

Transcripts related to transporters comprising of sucrose, sugar, sugar efflux, sugar phosphate exchanger, hexose, nitrate, GDP-mannose, aquaporins, vacuolar ATP synthase subunit C, vacuolar H^+^ ATP synthase subunit C, vacuolar H^+^ pyrophosphatase, vacuolar proton pumps, vacuolar targeting receptors, vacuolar protein sorting proteins (1, 13, 13A, 22, 25, 33, 36, 41, 55, DUF1162) and vacuolar H^+^- inorganic pyrophosphatase were found to be upregulated. A transcript was found to match the bacterial sugar transport system probably due to contaminating sequences. An abundance of ABC transporters could be observed in all DGEs.

#### Hormones

Auxin related transcripts were *auxin response factors*1, 3, 4, 5, 7, 9, 13, 15, 16, 17, 22, 23, 26, 27 and 31, *auxin responsive* proteins, auxin influx/efflux carriers, auxin transporters 1, 2, and *auxin binding protein* 4 were found. With respect to ethylene, 43 transcripts including *ethylene over-producer* like proteins, *ethylene responsive* transcription factors, elongation factors, calmodulin binding factors, element binding factors, small GTP binding proteins, ethylene receptors, and *ethylene insensitive* 2, and 3 proteins were found. Transcripts related to abscisic acid (ABA) and gibberellic acid (GA) and very few jasmonate and brassinosteroid related transcripts were found in the DEGs.

#### Organellar

Transcripts related to the chloroplast, notably chloroplastic group IIB intron splicing facilitator CRS2, alpha-glucan water dikinase, rubisco large subunit alpha binding, chloroplast post-illumination chlorophyll fluorescence increase protein, starch synthases II b and c, III, IV and starch branching and debranching (pullulanase and isoamylase) enzymes to name a few from the three DGEs. The ribosomal proteins were one of the most upregulated transcripts in all the DGEs comprising of nuclear, cytoplasmic, chloroplast and mitochondrial ribosome related functions especially of 30S, 40S, 50S, and 60S and acidic ribosomal transcripts.

#### Senescence/ripening/stress

Transcripts related to senescence including senescence-inducible chloroplast *stay*-*green* protein and *leaf senescence* proteins, *senescence*-*inducible chloroplast stay*-*green* protein, heat shock related transcripts of DNA and chloroplast, *wound inducible* protein, ripening ABA induced, autophagy, programmed cell death, cell death related protein, and *defender against cell death,* vascular death associated transcript were found. Transcripts were related to stress (light, water, heat, salt, ozone-responsive, bio-stress) and pathogenesis related transcripts, hypersensitive induced response proteins, 22 kDa drought inducible proteins, dehydrins and transcripts related to proline were found to be upregulated.

#### Flowering

Flowering related transcripts including pistil, pollen, immature pollen, flowering-time protein isoforms, phytochrome and *flowering time*, *flowering locus, GIGANTEA,* OVA4 ovule abortion 4*,* and *fertilization independent* were upregulated in high sugar genotypes. Proteins related to the egg apparatus, seed maturation, *shrunken seed* and seed starch branching enzyme related transcripts were upregulated. *HASTY 1* flower development, *agamous*-like MADS box AGL12, *photoperiod-independent early flowering* 1, *early flowering 3*, *flowering time control* FY, *luminidependens* are some of the flowering related transcripts found across the DEGs.

#### Signalling

Transcripts of signalling related to DNA damage, signal recognition, pollen, and integral membrane, *14–3-3* like proteins. Out of a large number of kinases, serine/threonine phosphatases, appeared to have a dominant role during sucrose accumulation. Also, it was observed that several signalling events can be inter related with others from the pattern of gene expression observed to be upregulated in high sugar genotypes (Additional file 9: Figure S5).

#### Fibre/cellulose

In the SoGI-DGE, transcripts matching with *fibre proteins* 11, 12, 15, 19 and 34 of cotton and *Hyacinthus* sp. were found. There was a transcript weakly similar to *cement protein* 3b from the marine worm *Phragmatopoma californica*. Vegetative and secondary cell wall proteins, cell wall hydrolases, cell envelope and cell shape, cell wall beta 1,3, endoglucanase cellulose synthases, bundle sheath cell specific proteins, 50 transcripts for cellulose synthases 2, 3, 4, 5, 6, E6, D3, A and 7, cellulose 1,4, beta-cellobiosidase were upregulated in high sugar genotypes. Also, transcripts of phenyl ammonia lyase (PAL), 6 caffeic acid-o-methyl transferase (COMT), caffeoyl CoA 3-O-methyl transferases (CCoAOMT), glutathione S-transferase, 6 dihydroxyacetone kinase, and transcripts related to chorismate, succinyl, cinnamoyl alcohol of shikimate pathway, caffeoylshikimate esterase, expansins A2 and A13, transcripts for vegetative cell wall, and secondary cell wall related transcripts were found.

#### Light/photosynthesis

Transcripts related to light/photosystem including light induced, light responsive proteins. *De-etiolated* 1, phytochrome, *rubisco* sub unit binding proteins, chloroplast post-illumination chlorophyll fluorescence increase protein, cryptochrome, photosystems I 700 and II 680 chlorophyll A apoprotein, photosystem reaction centre subunits II, III, VIII, IX, XI, 23 are few to mention. Interestingly, there were eight non-photosynthetic NADP-malic enzymes transcripts from *Zea mays* in SoGI-DGE. In SUGIT-DGE, transcripts of *CIRCADIAN TIMEKEEPER*, blue light photoreceptor PHR2, *negatively light regulated*, light-stress responsive one helix like, light inducible CPRF2 and *WEAK CHLOROPLAST MOVEMENTUNDER BLUE LIGHT* 1 like. Transcripts related to photosynthetic NDH subunit of subcomplex B chloroplastic, *light dependant short hypocotyls* 4 like, *high light induced chloroplastic* like, blue light photoreceptor PHR2 etc. were found in SAS-DGE. Nitrogen (N) related transcripts comprising of nitrogen utilization substrate protein, nitrogenase, nitrilase, *nitrate extrusion* proteins and nitrate reductase, bifunctional nitrilase nitrile hydratase NIT4A were up regulated in high sugar genotypes.

#### Uncharacterized

Interestingly, in SoGI-DGE, about 6552 transcripts were found to match the chromosomal regions of *Vitis vinifera* (SoGI annotation) which are whole genome shotgun sequences. In SUGIT-DGE, 243 transcripts were uncharacterized and in SAS-DGE, 320 transcripts were found to be uncharacterized.

### Down regulated transcripts in high sugar genotypes

The transcripts down regulated in high sugar genotypes included 17S, 18S, 26S, ribosomal RNA genes, cytochrome P450, and photosystem I 700, a stem specific transcript and leaf specific transcript from *Saccharum* hybrid cultivar, rRNA intron encoded homing endonuclease, zinc finger protein and uncharacterized transcripts in the three DGEs.

## Discussion

Two groups of genotypes, high sugar and low sugar, were formed based on the sugar content in terms of Brix as in sugarcane most of the soluble solids in the juice (70–91%) correspond to sucrose [12, 30]. Differential expression of genes was studied between the two groups and between top and bottom internodal samples (immature and mature) of the two groups. Therefore, gene expression changes were studied among high sugar top internode (HST), high sugar bottom internode (HSB), low sugar top internode (LST) and low sugar bottom internode (LSB)samples in various comparisons. Thus, the HST vs LST and HSB vs LSB were comparisons between the high and low sugar genotypes, whereas HST vs HSB and LST vs LSB were comparisons between top and bottom intermodal samples. For the DGE analyses, three databases were used as references individually wherein a large number of DEGs were identified from each. The databases were chosen to be specific for sugarcane. SoGI and SAS are derived from 26 different cDNA libraries [24] as a result, a large number of DEGs where obtained. The SUCEST database which encompasses SoGI and SAS is reported to cover >90% of the sugarcane genes [31]. The SUGIT database is essentially a long reads database sequenced using the latest Iso-Seq technology [17] which can further be used for refining the DEGs for isoform/allelic information. This database covers approximately 71% of the total predicted genes in sugarcane [17]. The common and unique transcripts from each database are not discussed further as the main objective of this paper was to find the DGE for sugar content. A subset of sucrose /sugar related DEGs were derived, which is interesting as several other studies on sucrose accumulation in sugarcane reported that sucrose related genes were less abundant or not expressed during the maturation stage [6, 12, 32]. There were approximately 70 transcripts related to sugar/sucrose in each DGE. Sucrose synthase (SuSy) and sucrose transporters (SuTs) were consistently found to be highly expressed in high sugar genotypes. Similar association was reported in [6, 8, 14]. The identity of the exact isoform of these two genes could not be found due to the varying annotations of the three databases used, which needs further studies. SuSy is reported to contribute to increasing the sink capacity, building cell wall materials and starch while sucrose transporters facilitate transportation of sucrose that leads to steady increase in sucrose content [30]. Further work on the isoforms/allelic expression of these genes would certainly be useful for understanding the finer details of their regulatory roles. The functioning of the two sucrose synthesis enzymes, SuSy and SPS and their regulation, has not yet been well demonstrated in sugarcane. SPS, sucrose non-fermenting related kinases, bidirectional sugar transporter SWEET, UDP-sugar pyrophosphorylase, *impaired sucrose induction* 1 -like proteins were the other genes that were consistently present at lower fold changes. Interestingly, an *invertase inhibitor* gene was found to be highly expressed in LST (13 folds) in LST vs LSB in SAS-DGE. Invertase inhibitors have been previously reported to be highly expressed during the sucrose accumulation stages in sugarcane [33].

In addition to the above genes, the gene expression pattern in our study reveals a clear association between different gene networks during sucrose accumulation similar to earlier reports [9, 12]. It is possible to make a direct parallel between sucrose content and gene expression levels for almost all the DEGs though the difference in the sugar content between the two groups is very narrow. Sucrose is a carbohydrate compound and was originally recognized only as an energy source for metabolism in plants but was recently shown to also function as a signalling molecule involved in regulation of various physiological processes in plants such as root growth, fruit development and ripening, and hypocotyl elongation [34]. Sugars serve as key components reflecting the plant’s energy status and, therefore, the ability to continuously sense sugar levels and control energy status is a key to survival and therefore transcript levels of thousands of genes respond to changing sugar levels [34]. Further, different sugars can have different regulatory roles in physiological processes, and the developmental stage of the plant further determines the response to sugars [35–37]. Recently, it was observed that glucose facilitates the juvenile to adult phase change in Arabidopsis by repressing microRNA (miRNA) 156 expression [38–40]. Consequently, mutants in sugar signalling or starch metabolism display an altered juvenile phase [38]. At high concentrations, sugars can induce meristem quiescence as observed in the arrest of development of seedlings germinated on high sugar levels [34]. Sugar induced quiescence of the stem can be seen through the expression of several transcripts for *no apical meristem* and *indeterminate spikelet* transcripts in addition to senescence related transcripts. Transcripts related to less abundant and lignocellulosic sugars identified in this study included xylose, trehalose, galactose, arabinose, fucose, mannose, taurine and the sugar alcohols inositol and sorbitol. The constant synthesis and breakdown of sucrose into its hexose components helps regulating various physiological events associated with these less abundant sugars and maintain a reserve for tackling any stimuli including the accumulation of sugar in the form of sucrose. It could be possible that breeding programmes for high sucrose genotypes have resulted in selection for these sugars (total sugars, in addition to sucrose) and gene expression changes of certain regulatory genes [15]. Therefore, diverse phenotypes may stem from multiple effects of sucrose and other sugars as signal and storage compounds when accumulated in various developmental and compartmental patterns resulting from differential gene expression and regulation.

The vacuole occupies as much as 90% of most mature cells and can accumulate and store sucrose, glucose and fructose and serves as a primary pool of free calcium ions in plant cells. Furthermore, the space-filling function of the vacuole is essential for cell growth, as the cell enlargement is mainly through the expansion of the vacuole rather than of the cytoplasm [41]. A vast majority of the differentially expressed gene transcripts were vacuole related including aquaporins, glucans related, aspartic proteinases, endopeptidases, ABC transporters, TIPs, V-ATP synthases, vacuolar protein sorting proteins, proton pumps, Ca^2+^ ATPases, calmodulins, that showed higher expression levels in high sugar genotypes. Further molecular characterization of vacuolar and tonoplast sugar transporters should advance our understanding of vacuole function, sugar transport and sugar accumulation in sugarcane.

Several transcripts related to plant defense, wounding, and disease were upregulated in high sugar genotypes together with the ripening and senescence related transcripts. Further, water stress and dehydration related gene transcripts were upregulated in the high sugar genotypes. Apart from the ripening and senescence related transcripts which indicate the physiological state of the stem, the up regulation of transcripts encoding plant disease resistance proteins suggests that the defense system of sugarcane was activated by high sugar levels which might contribute to protecting from the extreme stress caused by the high sucrose levels during the maturity stages. It may also create steep osmotic gradients between compartments with varying sucrose concentrations (more negative than −2.0 MPa during sucrose accumulation). The increased commitment to fibre synthesis in the maturing stem is evident in the upregulation of several fibre and cellulose related transcripts in the high sugar genotypes highlights the need to maintain the structure of the stem in conjunction with sucrose accumulation. These may act to restrict apoplastic movement of solutes between the vascular bundles and the sucrose-storage parenchyma cells [42]. Transcripts related to proline and glyoxalase were highly expressed in high sugar genotypes. The differential expression of genes related to fibre, cellulose and lignin synthesis shows that the osmotic regulation and structural maintenance as directed by the sugar levels. Though sucrose content in the sugarcane culm ranges from 14 to 42% of the culm dry weight [3], the majority of carbohydrates in sugarcane is lignocellulose, a major component in the cell wall. As cell elongation and sucrose accumulation ceases in the maturing sugarcane internodes, there is a major increase in cell wall thickening and lignification [43]. Cellulose accounts for about 42–43% in sugarcane and energy cane cultivars [44] and can be a prominent competing sink for carbon in sugarcane. Cellulose synthases 1, 2, 3, 4, 5, 6, 7, and 9 along with a novel transcript matching for a *cement protein* like gene that is upregulated in high sugar genotypes indicates that there are several aspects of sugarcane cell wall composition remain to be explored [45]. S-adenosylmethionine (SAM) produced by SAM-synthase is required as the methylation donor in lignin and suberin biosynthesis and secondary metabolism. It is also required as a precursor for SAM-decarboxylase, which is also up-regulated and important in polyamine synthesis, a response to osmotic stress. Elevated SPS activity is consistently correlated with high rates of cellulose synthesis and secondary wall deposition [46]. UDP-Glucose, apart from being the precursor for sucrose synthesis, is a nucleotide sugar central to diverse pathways of polysaccharide biosynthesis, leading to starch and cellulose, hemicellulose and callose synthesis. About 10 major monosaccharides in cell wall polymers are converted from glucose through UDP-Glucose related interconversion pathways. All UDP-Glucose related transcripts including UDP-Glucose dehydrogenases, pyrophosphorylases were upregulated in high sugar genotypes indicating a high correlation with sugar contents. Ethylene is often related to the lignification of plant tissues by increasing the expression of genes involved in the phenylpropanoid pathway [47]. This explains the parallel upregulation of cellulose synthases, ethylene related transcripts, as well as SPS in the DGEs. The mechanisms regulating cell wall biosynthesis and source-sink relations in sugarcane will be crucial constituents of any efforts to alter carbon partitioning between fibre and sugar in the culm. In addition, the alteration of cell wall biosynthesis genes in association with sucrose (Brix) content is an interesting indication of a correlation between these processes. Silencing or over-expression of some of these genes may lead to altered cell wall or increased sucrose content. Interestingly, when comparing two genotypes contrasting for lignin content, Vicentini et al. [45] found that a simple correlation between lignin content and differential expression of lignin genes is not always straightforward and most of the lignin biosynthetic genes did not show increased transcript levels in the high lignin genotype.

Sugar signals and the circadian clock are part of a complex network that controls floral transition. In sugarcane, sugar levels peak just before flowering induction. The signalling for senescence, arrest of apical growth, high sucrose levels and flowering induction are well coordinated. The upregulation of several flowering related genes like *flowering locus D*, pollen and pistil related transcripts in the high sugar genotypes clearly shows that the crop has attained its maximum sugar levels and was in a transition state to flowering though many are commercial cultivars that do not or flower rarely. Sugarcane has been selected for higher sugar content that involved strategies for delayed flowering and seed set, due to which a majority of sugarcane cultivars now are either sterile or the reproductive cycle has been delayed, or dormant for years [48]. Trehalose and its phosphate derivative trehalose-6- phosphate have recently gained importance as signalling molecules involved in carbon partitioning and also linking sugar status and diurnal rhythm to floral transition, in plants [49, 50]. For example, high sucrose and trehalose-6-phosphate (T6P) levels signal a cellular sugar abundance status [37, 51]. In addition to other sugar forms, the role of trehalose in sugarcane sucrose metabolism needs further studies as corroborated by the upregulation of several transcripts for trehalose phosphate synthase and trehalases.

Light interception and the *stay-green* trait are considered as major factors influencing the level of carbohydrates in the internodes [51]. Leaf angle is a genetic trait and higher sucrose yield in sweet sorghum can be achieved by genetic adjustment of leaf angles to optimum light interception. In addition, *stay-green* varieties of sweet sorghum were found to have higher stem sugar concentrations than senescing lines [52]. This may be due to the reduced need for re-mobilizing stem sucrose in addition to prolonged photosynthetic capacity [53]. Similarly, the upregulation of *stay-green* gene transcripts in the high sugar genotypes indicates an association between high sugar levels and higher photosynthetic capacity as the C4 enzymes are mainly localized in the chloroplasts. Further, high expression levels of photosynthetic, light harvesting, etiolation, starch, chlorophyll, gene transcripts were observed in high sugar genotypes. In addition, transcripts related to non- photosynthetic NADP malic enzymes [54] were upregulated in high sugar genotypes for which the functional significance is unknown in sugarcane. The rapid cycling of sugars in non-photosynthetic cells has been referred to as a ‘futile cycle’ [55] because of the continuous and simultaneous synthesis and degradation of sucrose. However, it is recognised that these cycles allow cells to respond in a highly sensitive manner to small changes in the balance between the supply of sucrose and the demand for carbon for respiration and biosynthesis and thus resulting in a strong sink [30]. This remobilisation of stored sucrose as a food supply results in rapid regrowth following stress or in germination of axillary buds of the internode [56]. Photosynthesis, growth and yield are strongly linked to N availability especially in C4 crops [57].The upregulation of N related transcripts in high sugar genotypes indicate that this is an ongoing process even if the crop has reached maturity.

**Fig. 6:**
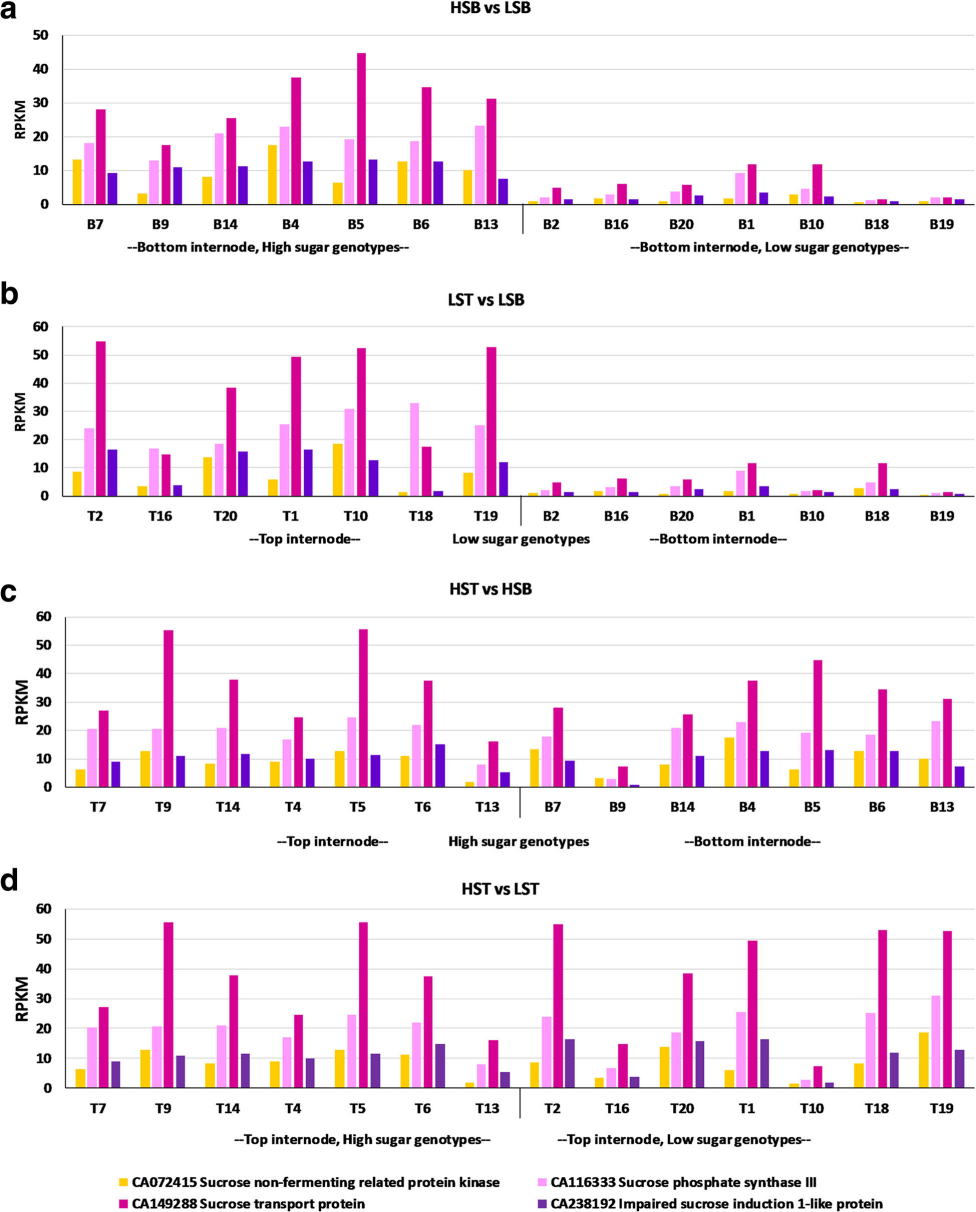
Graphical representation of the expression pattern of sucrose synthase transcripts in various comparisons. LST, low sugar top internode samples; LSB, low sugar bottom internode samples; HST, high sugar top internode samples; HSB, high sugar bottom internode samples; T-top tissue; B-bottom tissue. Shown here are the sucrose synthase (SuSy) transcripts from *Saccharum officinarum* gene indices, SoGI database, showing differential expression in top two comparisons. (**a**) HSB vs LSB; (**b**) LST vs LSB, while there is no differential expression in case of lower two comparisons (**c**) HST vs HSB; (**d**) HST vs LST at FDR<0.01. X-axis shows the genotypes while Y-axis represents RPKM values

**Fig. 7:**
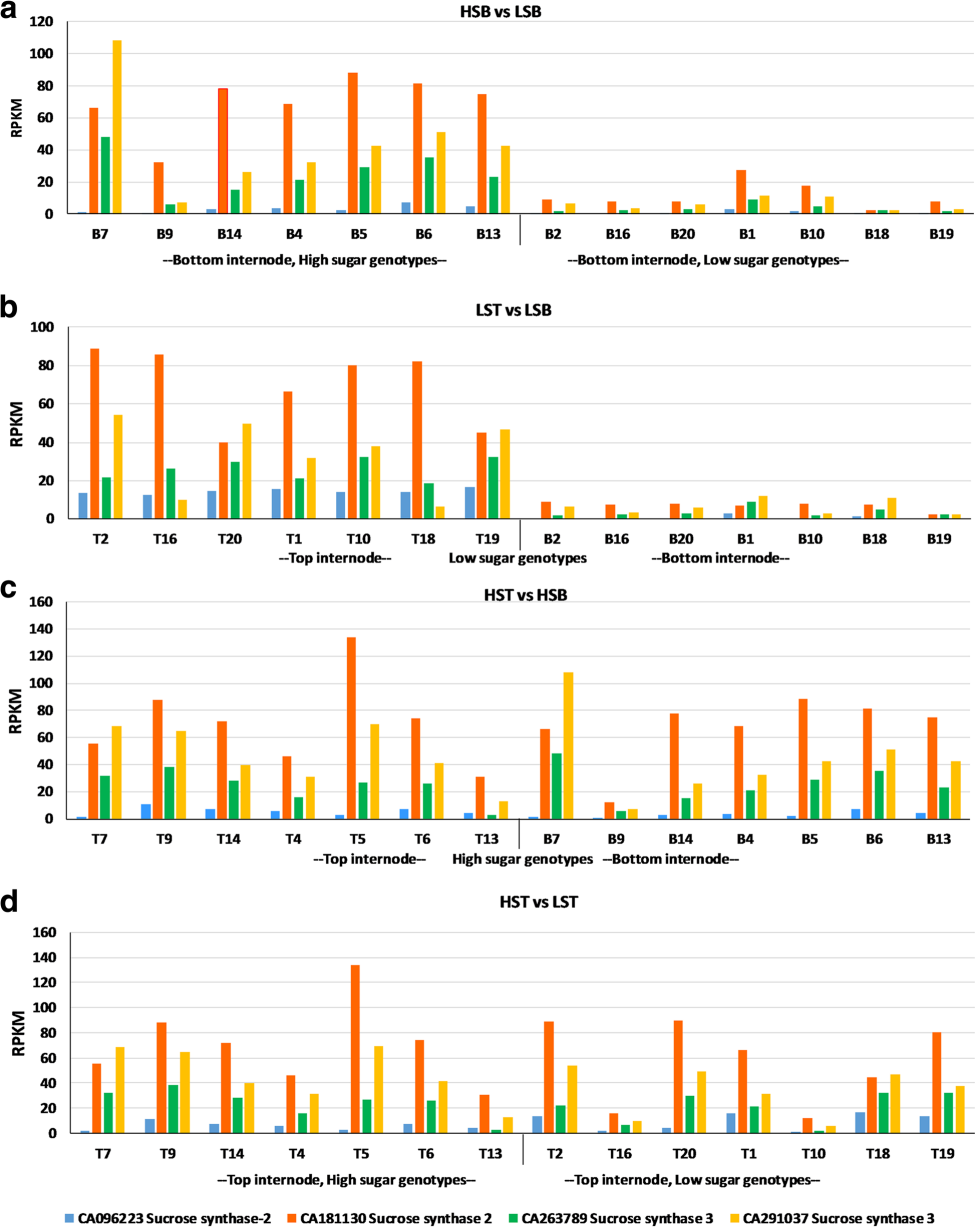
Graphical representation of the expression pattern of some of SuSy transcripts in various comparisons. LST, low sugar top internode sample; LSB, low sugar bottom internode sample; HST, high sugar top internode sample; HSB, high sugar bottom internode sample; T-top tissue; B-bottom tissue. Shown here are the sucrose phosphate synthase III, sucrose non-fermented related protein kinase, sucrose transport protein, impaired sucrose induction 1- like protein transcripts from *Saccharum officinarum* gene indices, SoGI database, showing differential expression in top two comparisons (**a**) HSB vs LSB; (**b**) LST vs LSB while there is no differential expression in lower two comparisons (**c**) HST vs HSB; (**d**) HST vs LST at FDR < 0.01. X-axis shows the genotypes while Y-axis represents RPKM values

The general cell related functions and growth, organellar and nuclear functions, biosynthetic pathways of pigments, amino acids, metabolites, hormonal signalling, transcription factors, various other transporters, proteins of transposons, root/stem/leaf related transcripts, were upregulated in the high sugar genotypes. The functions enriched in genes that are differentially expressed between different tissues in each comparison are consistent with the physiological changes associated with the development of that tissue, mainly sucrose content (Figs. 6 and 7). The absence of DEGs in HST vs LST suggests that the top internodes are metabolically active irrespective of their sucrose contents (i.e. high or low sugar genotype). The absence of sucrose related DEGs in HST vs HSB, where the top and bottom internodes of high sugar genotypes show almost similar expression patterns, indicates homogeneity for sucrose content throughout the culm. Further, the high sugar genotypes seem to invest in more fibre and cellulose as revealed by the nature of the transcripts that are differentially expressed between top and bottom internodes (Table 6). Meanwhile, a large number sucrose related DEGs in LST vs LSB shows that a gradient for sucrose exists in the low sugar genotypes. This observation can be inferred in two possible ways. One is that the low sugar genotypes have an active top internode compared to bottom leading to sucrose futile cycling, resulting in less accumulation or the other way could be that the bottom internodes have slowed down metabolically over time, reaching their physiological threshold levels of sucrose. The former is unlikely as there are no acid invertases (cell wall/ vacuolar) expression observed in the DEGs which are involved in the sucrose breakdown. The latter is likely to be the reason and the bottom internodes play a major role in the sucrose content of the genotypes. Also, the bottom internode of high sugar genotypes shows high expression of sucrose related genes. Feedback inhibition or post translational regulation could possibly be involved in the low sugar genotypes having higher expression of the sucrose related genes in the top internode and in turn having a low sugar content. In addition, the low sugar genotypes could also be late maturing genotypes, as some of them are introgression lines (other than the commercial hybrids) not having an established sugar profile or maturity indices yet (for e.g., fibre: sugar ratio). Many factors besides Brix, like ratoonability, vigour, softness, several resistance mechanisms, secondary metabolites, starch, etc., may also differ among the genotypes taken that remain to be evaluated. There were 7814 transcripts unique to HSB vs LSB, and 3667 transcripts unique to LST vs LSB (of the 34,476 DEGs in HSB vs LSB and 30,809 DEGs in LST vs LSB). These transcripts may indicate tissue specificity of the genes or their isoforms which is to be explored. When these unique transcripts were filtered for sucrose/sugar genes, SPS, SPP, SuSy, and sugar transporter genes were more specific to HSB vs LSB whereas, only some of the sugar transporter genes were specific to LST vs LSB (Additional file 8: Tables S24–28).

It was proposed that sucrose accumulation may be regulated by a network of genes induced during culm maturation that contribute to key physiological processes including sugar translocation/transport, fibre synthesis, membrane transport, vacuole development and function, and abiotic stress tolerance [9, 12]. We found a similar trend in this profiling study, in addition to a very high number of differentially expressed sucrose and sugar related transcripts that might help bridge missing links in the interlinking of biosynthetic pathways and their regulatory factors. It is to be studied if sucrose regulates the large number of genes or large number genes is required for controlling this trait. As sucrose emerges as a signalling molecule as seen in the recent studies [34, 50], the all-pervasive nature of this sugar is likely to regulate the growth and developmental processes of the plant. It can be speculated for the presence of master switches or the major regulatory genes of this trait as further genomic information is obtained in the future. Many novel genes, like caffeoyl shikimate esterase that was recently discovered in Arabidopsis and reported to be absent in sugarcane [44] where found in the upregulated transcripts in our study. Further mining of the transcriptomes would certainly lead to new targets and new aspects for sucrose synthesis and accumulation in sugarcane.

## Conclusion

The data reported here provide a comprehensive resource for sucrose related as well as culm maturation related studies in sugarcane. Further studies on a large data set with different developmental time points for genotypes contrasting for sugar content or energy canes that do not accumulate high levels of sugars should indicate targets for further biotechnological approaches. A dedicated analysis of transcription factors, and regulatory elements will further help understanding the complexity of the sugar network. Sucrose accumulation is very dynamic and unlike fruiting organs, the sugarcane culm is continuously exposed to every possible stimulus in the crop, soil and water continuum which results in a plethora of genes that are expressed at any point of time (approximately about 33,000). Although the present study identified more than 30,000 genes regulated and differentially expressed between high and low sugar genotypes, it is hard to pinpoint any particular group of genes or a gene to be responsible for the sucrose content and maintenance. Further, it is not possible for a gene to be lacking or not expressed in either of the groups as sucrose is a primary metabolite and principal transport sugar in sugarcane, which shows that the trait is quantitative and it is under transcriptional control. The machinery for sucrose synthesis is conserved across species and it is supposed that the complexity of sugarcane genome must play an important role in the sucrose levels that are observed in sugarcane. With multiple forms of each enzyme, with their own isoforms, various localizations, compartmentalized processes, the availability of large vacuoles and a unique stem morphology together contributes to the sugarcane stem sucrose content. Further, the availability of multiple isoforms or alleles gives the crop the advantage of buffering against any functional disruption which is the main reason for the instability of transformation events in sugarcane [58]. With these challenges in the sugarcane crop, a multitude of strategies are required for any genetic manipulation or for identification of regulatory genes for important traits particularly sucrose.

## Additional files

Additional file 1: Table S1a. Sugar profile of the genotypes taken for the study. **Table S1b** Quality Report of RNA Seq reads of samples used in the study. **Table S2** Genotypes and their transcriptome samples taken based on sugar content (Brix). **Table S3** Additional information with regard to genotypes selected. **Figure S1** Graphical representation of the sugar profiles of the genotypes selected for the study. (XLSX 2248 kb)

Additional file 2: Table S4. Complete list of DEGs in the SOGI-DGE. **Table S5** List of DEGs of SOGI HSB VS LSB. **Table S6** List of DEGs of SOGI HST VS HSB. (XLSX 8800 kb)

Additional file 3: Table S7. List of DEGs in the SUGIT, HST VS HSB. **Table S8** List of DEGs in the SUGIT, LST VS LSB. **Table S9** List of DEGs in the SUGIT, HST VS HSB. (XLSX 3817 kb)

Additional file 4: Table S10. List of DEGs in the SAS-DGE. **Table S11** List of DEGs in the SAS-DGE. **Table S12** List of DEGs in SAS-DGE (Excel workbook). (XLSX 5447 kb)

Additional file 5: Table S13. qPCR for selected transcripts and correlation analysis with RNA-Seq expression values. **Figure S2** Correlation analysis of RNA- Seq and qRT-PCR expression values for selected transcripts. (XLSX 28 kb)

Additional file 6: Table S14. DEGs of the experiment high sugar bottom vs low sugar bottom with SoGI database. **Table S15** DEGs in the experiment low sugar top vs low sugar bottom with SoGI database. **Table S16** DEGs in the experiment high sugar top vs high sugar bottom SoGI database. **Table S17** DEGs in high sugar bottom vs low sugar bottom with SUGIT database. **Table S18** DEGs obtained in High sugar top vs high sugar bottom with SUGIT database. **Table S19** DEGs obtained in low sugar top vs low sugar bottom with SUGIT database. **Table S20** DEGs in high sugar top vs high sugar bottom experiment with SAS database. **Table S21** DEGs in low sugar top vs low sugar bottom experiment with SAS database. **Table S22** DEGs in high sugar bottom vs low sugar bottom experiment with SAS database. (DOCX 45 kb)

Additional file 7: Figure S3. Functional classification of the DEGs obtained in three different DGEs using Mapman annotation. 1. High sugar top vs high sugar bottom internode samples (HST vs HSB), 2. Low sugar top vs low sugar bottom internode samples (LST vs LSB), 3. High sugar bottom vs low sugar bottom internode samples; a, d, g) *Saccharum officinarum* gene indices, SoGI; b, e, h) Sugarcane long read database, SLRD; c, f, i) Sugarcane assembled sequences, SAS. **Table S23** Functional classification of the DEGs obtained in three different DGEs using Mapman annotation. 1. High sugar top vs high sugar bottom internode samples (HST vs HSB), 2. Low sugar top vs low sugar bottom internode samples (LST vs LSB), 3. High sugar bottom vs low sugar bottom internode samples; a, d, g) *Saccharum officinarum* gene indices, SoGI; b, e, h) Sugarcane long read database, SLRD; c, f, i) Sugarcane assembled sequences, SAS. (XLSX 101 kb)

Additional file 8: Table S24. Common and unique transcripts between LST vs LSB and HSB vs LSB (SoGI-DGE). **Table S25** Common and unique transcripts between LST vs LSB and HSB vs LSB (SUGIT-DGE). **Table S26** Common and unique transcripts between LST vs LSB and HSB vs LSB (SAS-DGE). **Table S27** Unique transcripts (sucrose/sugar related) in HSB vs LSB (SoGI-DGE). **Table S28** Unique transcripts (sucrose/sugar related) in LST vs LSB (SoGI-DGE). (XLSX 3047 kb)

Additional file 9: Figure S4. Blast2GO and KEGG Mapping for DEGs in the SOGI-DGE with respect to starch and sucrose metabolism. **Figure S5** Sucrose emerges as a signalling molecule regulating most of the inter-linked plant functions in sugarcane. The gene expression pattern in culm tissue during sucrose accumulation in the genotypes studied reveals several networks of genes regulated by sucrose, and correlating with the sucrose content of the genotypes studied. (XLSX 469 kb)

## Abbreviations

ABA: Abscisic acid
BC: Back cross
BE: β-expansin
Bp: Basepair
CCoAOMT: Caffeoyl CoA 3-O-methyl transferases
CCoAR: Cinnamoyl CoA reductase
CCS: Commercial cane sugar
cDNA: Complementary DNA
CHS: Chalcone synthase
COMT: 6 caffeic acid-o-methyl transferase
DEGs: Differentially expressed genes
DGE: Differential gene expression
EST: Expressed sequence tag
FDR: False discovery rate
FE: Ferruloyl esterase
GA: Gibberellic acid
GDP: Guanosine diphosphate
GO: Gene ontology
GTP: Guanidine tri phosphate
HPC: High performance computing
HPLC: High performance liquid chromatography
HSB: High sugar bottom internode
HST: High sugar top internode
KEGG: Kyoto Encyclopedia of Genes and Genomes
LAC: Laccase
LSB: Low sugar bottom internode
LST: Low sugar top internode
MPa: Megapascal
mRNA: Messenger RNA
NADP: Nicotinamide adenosine diphosphate
NCBI: National Center for Biotechnology Information
NGS: Next generation sequencing
NIR: Near infrared
Nr database: Non-redundant database
ORF: Open reading frame
PAL: Phenyl ammonia lyase
PE: Paired end
qRT-PCR: Quantitative real-time polymerase chain reaction
RNA-seq: Ribonucleic acid sequencing
RPKM: Reads per kilobase per million mapped reads
SAM: S-adenosylmethionine
SAS: Sugarcane assembled sequences
SNP: Single nucleotide polymorphism
SoGI: *Saccharum officinarum* Gene Index
SPP: Sucrose phosphate phosphatase
SPS: Sucrose phosphate synthase
SPT: Sugar phosphate translocator
SRA: Sugar Research Australia
SSR: Simple sequence repeats or microsatellites
SUCEST: Sugarcane expressed sequence tag database
SUGIT: Sugarcane Iso-Seq transcriptome database
SuSy: Sucrose synthase
SuT: Sucrose transporter
TC: Terpene cyclase
TCA: Tricarboxylic acid
TSA: Transcriptome shot-gun assembly
UDP: Uridine diphosphate
V-ATP: Vacuolar adenosine triphosphate
