# Supplementary material for: Association of variation in the sugarcane transcriptome with sugar content
Source: BMC Genomics. 2017 Nov 25;18:909. doi: 10.1186/s12864-017-4302-5 (PMC5702190; doi:10.1186/s12864-017-4302-5)
Supplement: Supplementary file 6 — DEGs of the experiment high sugar bottom vs low sugar bottom with SoGI database. Table S15 DEGs in the experiment low sugar top vs low sugar bottom with SoGI database. Table S16 DEGs in the experiment high sugar top vs high sugar bottom SoGI database. Table S17 DEGs in high sugar bottom vs low sugar bottom with SUGIT database. Table S18 DEGs obtained in High sugar top vs high sugar bottom with SUGIT database. Table S19 DEGs obtained in low sugar top vs low sugar bottom with SUGIT database. Table S20 DEGs in high sugar top vs high sugar bottom experiment with SAS database. Table S21 DEGs in low sugar top vs low sugar bottom experiment with SAS database. Table S22 DEGs in high sugar bottom vs low sugar bottom experiment with SAS database. (DOCX 45 kb) [file 12864_2017_4302_MOESM6_ESM.docx]

**Table S14. DEGs of the experiment high sugar bottom vs low sugar bottom with SoGI database**

| Feature | ID | Fold | Weighted | FDR |
| --- | --- | --- | --- | --- |
| CA255667 | homologue to UniRef100_P49036 Cluster: Sucrose synthase 2; n=1; Zea mays | -28.85 | 25.72 | 0.01 |
| CA207180 | homologue to UniRef100_Q6PST5 Cluster: Sucrose transporter 2; n=1; Zea mays | -18.14 | 16.6 | 5.53E-03 |
| TC120829 | homologue to UniRef100_Q5TK93 Cluster: Sucrose synthase; n=1; Bambusa oldhamii | -12.2 | 10.98 | 0.01 |
| TC136397 | UniRef100_Q9LKR0 Cluster: Sucrose synthase-2; n=3; Saccharum | -10.97 | 9.93 | 2.56E-03 |
| CA243469 | homologue to UniRef100_P49036 Cluster: Sucrose synthase 2; n=1; Zea mays | -9.8 | 9.04 | 5.22E-03 |
| CA274886 | homologue to UniRef100_P93782 Cluster: Sucrose-Phosphate Synthase; n=1; Saccharum of | -8.97 | 8.06 | 2.68E-04 |
| TC145560 | UniRef100_Q6YLN4 Cluster: Sucrose synthase; n=1; Saccharum officinarum | -8.53 | 7.74 | 2.85E-03 |
| TC121981 | homologue to UniRef100_Q8L5H0 Cluster: Sucrose synthase 3; n=1; Zea mays | -8.42 | 7.65 | 1.88E-04 |
| TC113610 | UniRef100_Q4FCW1 Cluster: Sucrose phosphate phosphatase; n=1; Saccharum officinarum | -8.29 | 7.44 | 1.66E-03 |
| TC116268 | UniRef100_P93782 Cluster: Sucrose-Phosphate Synthase; n=1; Saccharum officinarum | -7.99 | 7.29 | 3.17E-05 |
| TC121542 | UniRef100_Q9LKR0 Cluster: Sucrose synthase-2; n=3; Saccharum | -7.73 | 6.95 | 3.03E-04 |
| TC146526 | homologue to UniRef100_Q6PST5 Cluster: Sucrose transporter 2; n=1; Zea mays | -7.62 | 6.93 | 4.57E-04 |
| CA072415 | homologue to UniRef100_A5HNE9 Cluster: Sucrose non-fermenting related protein kinase | -7.5 | 6.82 | 3.33E-05 |
| TC125025 | homologue to UniRef100_Q8L5H0 Cluster: Sucrose synthase 3; n=1; Zea mays | -7.39 | 6.72 | 5.71E-04 |
| TC133606 | homologue to UniRef100_Q8L5H0 Cluster: Sucrose synthase 3; n=1; Zea mays | -7.21 | 6.54 | 2.66E-04 |
| CA263789 | similar to UniRef100_Q8L5H0 Cluster: Sucrose synthase 3; n=1; Zea mays | -7.21 | 6.55 | 3.33E-04 |
| CA068686 | similar to UniRef100_Q6EZE8 Cluster: Sucrose-phosphate synthase; n=1; Triticum aesti | -7.15 | 6.49 | 1.75E-04 |
| CA291037 | homologue to UniRef100_Q8L5H0 Cluster: Sucrose synthase 3; n=1; Zea mays | -7.07 | 6.42 | 8.39E-03 |
| TC147386 | homologue to UniRef100_A5HNE9 Cluster: Sucrose non-fermenting related protein kinase | -6.95 | 6.32 | 3.60E-04 |
| TC137271 | homologue to UniRef100_Q8L5H0 Cluster: Sucrose synthase 3; n=1; Zea mays | -6.94 | 6.31 | 3.02E-04 |
| TC127787 | homologue to UniRef100_Q6PST5 Cluster: Sucrose transporter 2; n=1; Zea mays | -6.75 | 6.11 | 6.69E-05 |
| TC140141 | similar to UniRef100_Q5TJC3 Cluster: Impaired sucrose induction 1-like protein; n=2; | -6.53 | 5.96 | 4.84E-04 |
| CA142844 | similar to UniRef100_Q1WLP2 Cluster: Sucrose transporter type 2; n=1; Manihot escule | -6.46 | 5.83 | 8.54E-05 |
| TC128277 | UniRef100_Q5U871 Cluster: Sucrose transporter; n=1; Saccharum hybrid cultivar | -6.16 | 5.56 | 2.46E-05 |
| TC113476 | UniRef100_A9QVI4 Cluster: Sucrose phosphate synthase II; n=1; Saccharum officinarum | -6.15 | 5.56 | 1.65E-05 |
| CA181130 | homologue to UniRef100_P49036 Cluster: Sucrose synthase 2; n=1; Zea mays | -5.9 | 5.35 | 3.99E-06 |
| TC141576 | homologue to UniRef100_Q4FCW1 Cluster: Sucrose phosphate phosphatase; n=1; Saccharum | -5.83 | 5.29 | 5.72E-05 |
| TC138783 | similar to UniRef100_Q4LEV1 Cluster: Sucrose synthase; n=1; Potamogeton distinctus | -5.81 | 5.31 | 1.38E-05 |
| TC131675 | UniRef100_A9UFX6 Cluster: Sucrose phosphate synthase III; n=1; Saccharum officinarum | -5.74 | 5.21 | 2.64E-06 |
| TC117267 | homologue to UniRef100_A9UFX6 Cluster: Sucrose phosphate synthase III; n=1; Saccharu | -5.73 | 5.18 | 4.02E-06 |
| CA240368 | homologue to UniRef100_A5HNE9 Cluster: Sucrose non-fermenting related protein kinase | -5.69 | 5.21 | 2.88E-04 |
| TC119662 | homologue to UniRef100_A5HNE9 Cluster: Sucrose non-fermenting related protein kinase | -5.69 | 5.15 | 1.84E-05 |
| TC117483 | UniRef100_P93783 Cluster: Sucrose-phosphate synthase; n=1; Saccharum officinarum | -5.52 | 5 | 9.35E-06 |
| TC136732 | homologue to UniRef100_Q6J2T0 Cluster: Sucrose transporter SUT4; n=1; Zea mays | -5.49 | 4.99 | 8.38E-06 |
| CA209543 | homologue to UniRef100_P49036 Cluster: Sucrose synthase 2; n=1; Zea mays | -5.42 | 4.91 | 0.01 |
| TC140637 | similar to UniRef100_Q9LKW4 Cluster: Sucrose-phosphate synthase; n=1; Hordeum vulgar | -5.36 | 4.87 | 1.19E-04 |
| TC140795 | UniRef100_Q4FCW1 Cluster: Sucrose phosphate phosphatase; n=1; Saccharum officinarum | -5.35 | 4.88 | 1.71E-04 |
| TC119164 | homologue to UniRef100_A5HNE9 Cluster: Sucrose non-fermenting related protein kinase | -5.28 | 4.78 | 4.60E-06 |
| TC137871 | homologue to UniRef100_Q6GUI0 Cluster: Sucrose transport protein; n=1; Zea mays | -5.16 | 4.68 | 2.55E-05 |
| CA116333 | similar to UniRef100_A9UFX6 Cluster: Sucrose phosphate synthase III; n=1; Saccharum | -4.94 | 4.48 | 1.60E-05 |
| CA211785 | homologue to UniRef100_A9UFX6 Cluster: Sucrose phosphate synthase III; n=1; Saccharu | -4.9 | 4.48 | 3.76E-06 |
| CA238192 | similar to UniRef100_Q5TJC3 Cluster: Impaired sucrose induction 1-like protein; n=2; | -4.85 | 4.4 | 1.61E-04 |
| TC141871 | UniRef100_Q6YLN4 Cluster: Sucrose synthase; n=1; Saccharum officinarum | -4.83 | 4.36 | 6.55E-04 |
| TC120090 | similar to UniRef100_A9UFX5 Cluster: Sucrose phosphate synthase III; n=1; Saccharum | -4.79 | 4.34 | 9.21E-05 |
| CA149288 | homologue to UniRef100_Q6GUI0 Cluster: Sucrose transport protein; n=1; Zea mays | -4.77 | 4.33 | 1.86E-05 |
| TC145416 | homologue to UniRef100_A9QVI4 Cluster: Sucrose phosphate synthase II; n=1; Saccharum | -4.5 | 4.04 | 1.30E-03 |
| CA289856 | homologue to UniRef100_Q4FCW1 Cluster: Sucrose phosphate phosphatase; n=1; Saccharum | -4.48 | 4.08 | 2.52E-04 |
| TC148368 | homologue to UniRef100_A5HNE9 Cluster: Sucrose non-fermenting related protein kinase | -4.31 | 3.9 | 2.06E-03 |
| CA267680 | similar to UniRef100_Q9M1T1 Cluster: Sugar-phosphate isomerase-like protein; n=1; Arabidopsis thaliana | -17.31 | 15.59 | 4.84E-03 |
| TC112923 | homologue to UniRef100_Q41855 Cluster: Sugar-starvation induced protein; n=1; Zea mays | -15.38 | 13.92 | 7.76E-03 |
| CA204972 | similar to UniRef100_A4GXC9 Cluster: Sugar transporter protein; n=1; Ananas comosus | -10.53 | 9.51 | 2.86E-05 |
| TC153302 | similar to UniRef100_Q0VZ62 Cluster: ADP-sugar diphosphatase; n=1; Solanum tuberosum | -9.15 | 8.33 | 7.30E-05 |
| TC146639 | homologue to UniRef100_A2YGP6 Cluster: UDP-sugar pyrophosphorylase; n=1; Oryza sativa Indica Group | -8.39 | 7.65 | 3.65E-04 |
| TC126082 | similar to UniRef100_Q0VZ62 Cluster: ADP-sugar diphosphatase; n=1; Solanum tuberosum | -8.07 | 7.37 | 3.32E-04 |
| TC133377 | similar to UniRef100_Q2MGS1 Cluster: Glycosyltransferase sugar-binding region containing DXD motif; Alpha 1,4-glycosyltransferase conserved region; n=1; Medicago truncatula | -7.87 | 7.04 | 1.59E-03 |
| TC144527 | similar to UniRef100_Q2MGS1 Cluster: Glycosyltransferase sugar-binding region containing DXD motif; Alpha 1,4-glycosyltransferase conserved region; n=1; Medicago truncatula | -7.51 | 6.89 | 3.97E-06 |
| DV635495 | similar to UniRef100_Q8GTR0 Cluster: Sugar transporter; n=1; Citrus unshiu | -7.49 | 6.82 | 2.19E-06 |
| CA270057 | similar to UniRef100_A4GXC9 Cluster: Sugar transporter protein; n=1; Ananas comosus | -7.28 | 6.59 | 1.07E-03 |
| TC133843 | similar to UniRef100_A4GXC9 Cluster: Sugar transporter protein; n=1; Ananas comosus | -7.08 | 6.4 | 5.26E-04 |
| TC148606 | similar to UniRef100_A4GXC9 Cluster: Sugar transporter protein; n=1; Ananas comosus | -6.89 | 6.27 | 1.29E-04 |
| CA258700 | similar to UniRef100_A2CCR6 Cluster: Possible sugar transferase; n=1; Prochlorococcus marinus str. MIT 9303 | -6.69 | 6.11 | 1.04E-04 |
| CA136361 | similar to UniRef100_A2YGP6 Cluster: UDP-sugar pyrophosphorylase; n=1; Oryza sativa Indica Group | -6.48 | 5.84 | 0.01 |
| TC129699 | similar to UniRef100_A4GXC9 Cluster: Sugar transporter protein; n=1; Ananas comosus | -6.25 | 5.68 | 9.18E-05 |
| TC132436 | similar to UniRef100_A4GXC8 Cluster: Sugar transporter protein; n=1; Ananas comosus | -6.17 | 5.63 | 9.52E-06 |
| CA233504 | homologue to UniRef100_Q58CV5 Cluster: Sugar phosphate exchanger 2; n=1; Bos taurus | -6.15 | 5.57 | 1.68E-03 |
| DV731069 | similar to UniRef100_Q9KBQ9 Cluster: Sugar transport system; n=1; Bacillus halodurans | -6.08 | 5.48 | 7.66E-04 |
| TC131469 | similar to UniRef100_A2YGP6 Cluster: UDP-sugar pyrophosphorylase; n=1; Oryza sativa Indica Group | -5.78 | 5.24 | 4.86E-05 |
| CA178392 | similar to UniRef100_A4GXC8 Cluster: Sugar transporter protein; n=1; Ananas comosus | -5.73 | 5.22 | 9.35E-03 |
| TC149691 | similar to UniRef100_A4GXC8 Cluster: Sugar transporter protein; n=1; Ananas comosus | -5.64 | 5.12 | 3.11E-03 |
| CA257972 | similar to UniRef100_A4GXC8 Cluster: Sugar transporter protein; n=1; Ananas comosus | -5.32 | 4.81 | 5.05E-05 |
| TC146044 | similar to UniRef100_Q3ECP7 Cluster: Sugar transporter ERD6-like 5; n=1; Arabidopsis thaliana | -5.21 | 4.72 | 3.08E-06 |
| TC129039 | similar to UniRef100_A4FQ83 Cluster: Probable sugar efflux transporter, MFS superfamily; n=1; Saccharopolyspora erythraea NRRL 2338 | -4.92 | 4.46 | 1.24E-04 |
| BU925715 | similar to UniRef100_Q8GTR0 Cluster: Sugar transporter; n=1; Citrus unshiu | -4.58 | 4.16 | 1.90E-03 |
| CA109974 | homologue to UniRef100_Q8GT51 Cluster: Sugar transporter; n=1; Hordeum vulgare | -4.05 | 3.66 | 2.87E-04 |

**Table S15. DEGs in the experiment low sugar top vs low sugar bottom**

| Feature | ID | Fold | Weighted | FDR |
| --- | --- | --- | --- | --- |
| TC123316 | homologue to UniRef100_P49036 Cluster: Sucrose synthase 2; n=1; Zea mays | -19.2 | 17.13 | 0.01 |
| CA138819 | UniRef100_Q5U871 Cluster: Sucrose transporter; n=1; Saccharum hybrid cultivar | -18.57 | 16.81 | 0.01 |
| TC148821 | UniRef100_Q5U871 Cluster: Sucrose transporter; n=1; Saccharum hybrid cultivar | -15.99 | 14.47 | 2.42E-03 |
| TC128277 | UniRef100_Q5U871 Cluster: Sucrose transporter; n=1; Saccharum hybrid cultivar | -15.83 | 14.08 | 1.11E-03 |
| TC124887 | homologue to UniRef100_P49036 Cluster: Sucrose synthase 2; n=1; Zea mays | -14.57 | 13.27 | 0.01 |
| TC119654 | homologue to UniRef100_Q9LKR0 Cluster: Sucrose synthase-2; n=3; Saccharum | -13.85 | 12.35 | 0.01 |
| TC145560 | UniRef100_Q6YLN4 Cluster: Sucrose synthase; n=1; Saccharum officinarum | -12.24 | 10.95 | 1.77E-03 |
| TC121542 | UniRef100_Q9LKR0 Cluster: Sucrose synthase-2; n=3; Saccharum | -11.67 | 10.44 | 1.00E-03 |
| CA096223 | homologue to UniRef100_Q9LKR0 Cluster: Sucrose synthase-2; n=3; Saccharum | -10.9 | 9.78 | 4.97E-03 |
| TC141871 | UniRef100_Q6YLN4 Cluster: Sucrose synthase; n=1; Saccharum officinarum | -9.19 | 8.25 | 1.41E-03 |
| TC146526 | homologue to UniRef100_Q6PST5 Cluster: Sucrose transporter 2; n=1; Zea mays | -8.61 | 7.79 | 1.50E-03 |
| CA240368 | homologue to UniRef100_A5HNE9 Cluster: Sucrose non-fermenting related protein kinase | -8.31 | 7.51 | 2.30E-03 |
| TC127787 | homologue to UniRef100_Q6PST5 Cluster: Sucrose transporter 2; n=1; Zea mays | -7.88 | 7.12 | 7.80E-03 |
| TC147386 | homologue to UniRef100_A5HNE9 Cluster: Sucrose non-fermenting related protein kinase | -7.85 | 7.18 | 1.13E-03 |
| TC141576 | homologue to UniRef100_Q4FCW1 Cluster: Sucrose phosphate phosphatase; n=1; Saccharum | -7.69 | 6.98 | 7.81E-04 |
| TC140141 | similar to UniRef100_Q5TJC3 Cluster: Impaired sucrose induction 1-like protein; n=2; | -7.14 | 6.47 | 2.07E-03 |
| TC136732 | homologue to UniRef100_Q6J2T0 Cluster: Sucrose transporter SUT4; n=1; Zea mays | -7.08 | 6.35 | 1.02E-03 |
| TC137871 | homologue to UniRef100_Q6GUI0 Cluster: Sucrose transport protein; n=1; Zea mays | -6.66 | 5.99 | 7.74E-04 |
| TC113476 | UniRef100_A9QVI4 Cluster: Sucrose phosphate synthase II; n=1; Saccharum officinarum | -6.44 | 5.78 | 9.73E-04 |
| CA072415 | homologue to UniRef100_A5HNE9 Cluster: Sucrose non-fermenting related protein kinase | -6.38 | 5.75 | 7.52E-03 |
| TC138783 | similar to UniRef100_Q4LEV1 Cluster: Sucrose synthase; n=1; Potamogeton distinctus | -6.37 | 5.74 | 1.72E-03 |
| TC140637 | similar to UniRef100_Q9LKW4 Cluster: Sucrose-phosphate synthase; n=1; Hordeum vulgar | -6.24 | 5.61 | 2.28E-03 |
| CA149288 | homologue to UniRef100_Q6GUI0 Cluster: Sucrose transport protein; n=1; Zea mays | -6.17 | 5.53 | 8.90E-04 |
| TC119164 | homologue to UniRef100_A5HNE9 Cluster: Sucrose non-fermenting related protein kinase | -6.09 | 5.45 | 1.04E-03 |
| CA263789 | similar to UniRef100_Q8L5H0 Cluster: Sucrose synthase 3; n=1; Zea mays | -5.9 | 5.28 | 2.65E-03 |
| TC121981 | homologue to UniRef100_Q8L5H0 Cluster: Sucrose synthase 3; n=1; Zea mays | -5.87 | 5.28 | 3.06E-03 |
| CA238192 | similar to UniRef100_Q5TJC3 Cluster: Impaired sucrose induction 1-like protein; n=2; | -5.72 | 5.15 | 1.29E-03 |
| TC140795 | UniRef100_Q4FCW1 Cluster: Sucrose phosphate phosphatase; n=1; Saccharum officinarum | -5.63 | 5.11 | 1.71E-03 |
| TC117267 | homologue to UniRef100_A9UFX6 Cluster: Sucrose phosphate synthase III; n=1; Saccharu | -5.61 | 5.04 | 9.44E-04 |
| TC117483 | UniRef100_P93783 Cluster: Sucrose-phosphate synthase; n=1; Saccharum officinarum | -5.6 | 5.03 | 9.74E-04 |
| TC120090 | similar to UniRef100_A9UFX5 Cluster: Sucrose phosphate synthase III; n=1; Saccharum | -5.43 | 4.87 | 1.55E-03 |
| CA291037 | homologue to UniRef100_Q8L5H0 Cluster: Sucrose synthase 3; n=1; Zea mays | -5.4 | 4.83 | 1.86E-03 |
| TC145416 | homologue to UniRef100_A9QVI4 Cluster: Sucrose phosphate synthase II; n=1; Saccharum | -5.39 | 4.88 | 1.70E-03 |
| CA116333 | similar to UniRef100_A9UFX6 Cluster: Sucrose phosphate synthase III; n=1; Saccharum | -5.26 | 4.72 | 1.93E-03 |
| TC119662 | homologue to UniRef100_A5HNE9 Cluster: Sucrose non-fermenting related protein kinase | -5.24 | 4.69 | 4.91E-03 |
| TC125025 | homologue to UniRef100_Q8L5H0 Cluster: Sucrose synthase 3; n=1; Zea mays | -5.15 | 4.62 | 2.80E-03 |
| TC131675 | UniRef100_A9UFX6 Cluster: Sucrose phosphate synthase III; n=1; Saccharum officinarum | -5.14 | 4.6 | 2.35E-03 |
| TC137271 | homologue to UniRef100_Q8L5H0 Cluster: Sucrose synthase 3; n=1; Zea mays | -5.11 | 4.59 | 1.84E-03 |
| TC133606 | homologue to UniRef100_Q8L5H0 Cluster: Sucrose synthase 3; n=1; Zea mays | -5.07 | 4.54 | 1.81E-03 |
| CA181130 | homologue to UniRef100_P49036 Cluster: Sucrose synthase 2; n=1; Zea mays | -5.01 | 4.48 | 2.96E-03 |
| CA211785 | homologue to UniRef100_A9UFX6 Cluster: Sucrose phosphate synthase III; n=1; Saccharu | -5.01 | 4.54 | 9.16E-04 |
| CA289856 | homologue to UniRef100_Q4FCW1 Cluster: Sucrose phosphate phosphatase; n=1; Saccharum | -4.99 | 4.52 | 1.02E-03 |
| TC148368 | homologue to UniRef100_A5HNE9 Cluster: Sucrose non-fermenting related protein kinase | -4.96 | 4.43 | 0.01 |
| CA267680 | similar to UniRef100_Q9M1T1 Cluster: Sugar-phosphate isomerase-like protein; n=1; Arabidopsis thaliana | -24.39 | 22.16 | 2.84E-03 |
| TC149390 | similar to UniRef100_A4GXC8 Cluster: Sugar transporter protein; n=1; Ananas comosus | -11.06 | 9.99 | 8.84E-03 |
| TC150523 | similar to UniRef100_Q2MGS1 Cluster: Glycosyltransferase sugar-binding region containing DXD motif; Al | -11.03 | 9.97 | 0.01 |
| TC144527 | similar to UniRef100_Q2MGS1 Cluster: Glycosyltransferase sugar-binding region containing DXD motif; Al | -10.67 | 9.59 | 5.34E-03 |
| TC146639 | homologue to UniRef100_A2YGP6 Cluster: UDP-sugar pyrophosphorylase; n=1; Oryza sativa Indica Group | -10.45 | 9.53 | 8.92E-04 |
| CA227097 | weakly similar to UniRef100_A6WFD5 Cluster: Sugar transporter; n=1; Kineococcus radiotolerans SRS30216 | -9.13 | 8.3 | 9.18E-03 |
| TC140629 | similar to UniRef100_A4GXC8 Cluster: Sugar transporter protein; n=1; Ananas comosus | -9.11 | 8.34 | 1.50E-03 |
| CA178392 | similar to UniRef100_A4GXC8 Cluster: Sugar transporter protein; n=1; Ananas comosus | -8.55 | 7.73 | 5.18E-03 |
| TC153302 | similar to UniRef100_Q0VZ62 Cluster: ADP-sugar diphosphatase; n=1; Solanum tuberosum | -8.41 | 7.62 | 7.81E-04 |
| CA136361 | similar to UniRef100_A2YGP6 Cluster: UDP-sugar pyrophosphorylase; n=1; Oryza sativa Indica Group | -7.43 | 6.74 | 4.96E-03 |
| DV731069 | similar to UniRef100_Q9KBQ9 Cluster: Sugar transport system; n=1; Bacillus halodurans | -7.38 | 6.6 | 3.93E-03 |
| DV635495 | similar to UniRef100_Q8GTR0 Cluster: Sugar transporter; n=1; Citrus unshiu | -7.24 | 6.5 | 2.15E-03 |
| TC131469 | similar to UniRef100_A2YGP6 Cluster: UDP-sugar pyrophosphorylase; n=1; Oryza sativa Indica Group | -7.02 | 6.42 | 7.85E-04 |
| CA270057 | similar to UniRef100_A4GXC9 Cluster: Sugar transporter protein; n=1; Ananas comosus | -6.97 | 6.27 | 9.15E-03 |
| TC133843 | similar to UniRef100_A4GXC9 Cluster: Sugar transporter protein; n=1; Ananas comosus | -6.81 | 6.16 | 7.89E-03 |
| CA257972 | similar to UniRef100_A4GXC8 Cluster: Sugar transporter protein; n=1; Ananas comosus | -6.71 | 6.07 | 1.00E-03 |
| TC132436 | similar to UniRef100_A4GXC8 Cluster: Sugar transporter protein; n=1; Ananas comosus | -6.36 | 5.75 | 9.37E-04 |
| TC148606 | similar to UniRef100_A4GXC9 Cluster: Sugar transporter protein; n=1; Ananas comosus | -5.93 | 5.33 | 0.01 |
| TC146044 | similar to UniRef100_Q3ECP7 Cluster: Sugar transporter ERD6-like 5; n=1; Arabidopsis thaliana | -5.84 | 5.23 | 9.77E-04 |
| CA233504 | homologue to UniRef100_Q58CV5 Cluster: Sugar phosphate exchanger 2; n=1; Bos taurus | -5.74 | 5.18 | 5.88E-03 |
| CA109974 | homologue to UniRef100_Q8GT51 Cluster: Sugar transporter; n=1; Hordeum vulgare | -5.65 | 5.04 | 3.02E-03 |
| TC149691 | similar to UniRef100_A4GXC8 Cluster: Sugar transporter protein; n=1; Ananas comosus | -5.49 | 4.93 | 5.64E-03 |
| CA258700 | similar to UniRef100_A2CCR6 Cluster: Possible sugar transferase; n=1; Prochlorococcus marinus str. MIT | -5.46 | 4.96 | 3.94E-03 |
| BU925715 | similar to UniRef100_Q8GTR0 Cluster: Sugar transporter; n=1; Citrus unshiu | -5.4 | 4.85 | 3.01E-03 |
| TC129039 | similar to UniRef100_A4FQ83 Cluster: Probable sugar efflux transporter, MFS superfamily; n=1; Saccharo | -5.01 | 4.49 | 5.52E-03 |

**Table S16. DEGs in the experiment high sugar top vs high sugar bottom**

| Feature | ID | Fold | Weighted | FDR |
| --- | --- | --- | --- | --- |
| TC114623 | similar to UniRef100_Q0IT98 Cluster: Os11g0286800 protein; n=1; Oryza sativa Japonica Group | -18.58 | 18.7 | 7.51E-03 |
| TC125737 | similar to UniRef100_Q8VXG7 Cluster: Phenylalanine ammonia-lyase; n=1; Zea mays | -16.27 | 16.33 | 4.08E-03 |
| TC131133 | UniRef100_Q9SBL4 Cluster: Chalcone synthase 5; n=1; Sorghum bicolor | -13.79 | 13.91 | 4.24E-05 |
| CA207335 | similar to UniRef100_O82055 Cluster: Cinnamoyl-CoA reductase; n=1; Saccharum officinarum | -12.11 | 12.23 | 5.19E-03 |
| CA212197 | weakly similar to UniRef100_Q6IW99 Cluster: Beta-amyrin synthase; n=1; Avena prostrata | -11.85 | 12.04 | 0.01 |
| CA275219 | similar to UniRef100_A9TWY3 Cluster: Predicted protein; n=1; Physcomitrella patens subsp. patens | -6.52 | 6.49 | 0.01 |
| TC112853 | homologue to UniRef100_A2IBN5 Cluster: Phenylalanine ammonia-lyase; n=1; Saccharum officinarum | -6.05 | 6.1 | 4.16E-03 |
| CA113829 | homologue to UniRef100_Q9M7F3 Cluster: LIM transcription factor homolog; n=1; Zea mays | -5.75 | 5.76 | 7.51E-03 |
| TC142386 | similar to UniRef100_Q10PZ6 Cluster: Expressed protein; n=1; Oryza sativa Japonica Group | -5.72 | 5.72 | 5.19E-03 |
| CA147028 | homologue to UniRef100_A8WET3 Cluster: ZCN26; n=1; Zea mays | -5.7 | 5.69 | 1.06E-03 |
| TC148510 | homologue to UniRef100_Q9M7F3 Cluster: LIM transcription factor homolog; n=1; Zea mays | -5.6 | 5.63 | 6.04E-03 |
| CA194738 | similar to UniRef100_Q10PZ6 Cluster: Expressed protein; n=1; Oryza sativa Japonica Group | -5.57 | 5.56 | 8.82E-06 |
| CA065021 | similar to UniRef100_Q10PZ6 Cluster: Expressed protein; n=1; Oryza sativa Japonica Group | -5.47 | 5.46 | 1.56E-04 |
| CA065092 | homologue to UniRef100_Q1MXY7 Cluster: Universal stress protein family protein; n=1; Oceanobacter sp. RED65 | -5.43 | 5.44 | 1.48E-09 |
| TC120860 | similar to UniRef100_Q10PZ6 Cluster: Expressed protein; n=1; Oryza sativa Japonica Group | -5.38 | 5.4 | 3.93E-08 |
| TC125777 |  | -5.24 | 5.27 | 8.82E-06 |
| TC113904 | similar to UniRef100_Q0D8A1 Cluster: Os07g0174700 protein; n=1; Oryza sativa Japonica Group | -5.14 | 5.14 | 8.01E-03 |
| TC120814 | homologue to UniRef100_Q9M7F3 Cluster: LIM transcription factor homolog; n=1; Zea mays | -5.11 | 5.19 | 1.68E-04 |
| TC124516 | homologue to UniRef100_Q6Q297 Cluster: 4-coumarate coenzyme A ligase; n=1; Zea mays | -4.59 | 4.61 | 4.16E-03 |
| CA184462 |  | -4.52 | 4.58 | 8.52E-03 |
| CA253551 | homologue to UniRef100_Q0J9N6 Cluster: Os04g0640600 protein; n=1; Oryza sativa Japonica Group | -4.02 | 4.03 | 5.92E-03 |
| CF571583 | UniRef100_Q7NDY7 Cluster: Gll4095 protein; n=1; Gloeobacter violaceus | -3.79 | 3.85 | 0.01 |
| TC135370 |  | -3.77 | 3.76 | 0.01 |
| TC115208 | similar to UniRef100_Q0IPT6 Cluster: Os12g0169100 protein; n=1; Oryza sativa Japonica Group | -3.74 | 3.73 | 8.83E-03 |
| TC146921 | similar to UniRef100_A6MZE9 Cluster: Minor allergen alt a7; n=3; Oryza sativa | -3.61 | 3.6 | 5.31E-04 |
| TC137240 | weakly similar to UniRef100_Q94CU5 Cluster: Serine/threonine-protein kinase Nek5; n=1; Oryza sativa Japonica Group | -3.5 | 3.52 | 0.01 |
| CF574802 |  | -3.23 | 3.23 | 2.54E-04 |
| CA205661 | similar to UniRef100_A7NWE7 Cluster: Chromosome chr5 scaffold_2, whole genome shotgun sequence; n=1; Vitis vinifera | -3.04 | 3.04 | 7.17E-04 |
| TC147318 | weakly similar to UniRef100_A7PAI1 Cluster: Chromosome chr14 scaffold_9, whole genome shotgun sequence; n=1; Vitis vinifera | -2.77 | 2.76 | 0.01 |
| CA168645 | weakly similar to UniRef100_A7NWE7 Cluster: Chromosome chr5 scaffold_2, whole genome shotgun sequence; n=1; Vitis vinifera | -2.56 | 2.59 | 5.19E-03 |
| TC152422 | weakly similar to UniRef100_A7NWE7 Cluster: Chromosome chr5 scaffold_2, whole genome shotgun sequence; n=1; Vitis vinifera | -2.36 | 2.39 | 7.51E-03 |

**Table S17. DEGs in high sugar bottom vs low sugar bottom with SUGIT database**

| Feature ID | Length (bp) | Description | Fold Change (original values) | Weighted proportions fold change | FDR p-value correction |
| --- | --- | --- | --- | --- | --- |
| c98328f1p02743 | 2754 | B9VAS9_SORBI Sucrose synthase OS=Sorghum bicolor GN=SUSY2 PE=3 SV=1 | -10.46 | 8.86 | 3.32E-03 |
| c32435f3p21876 | 1880 | A5HNE9_SORBI Sucrose non-fermenting related kinase 1b OS=Sorghum bicolor PE=2 SV=1 | -8.47 | 7.19 | 8.64E-03 |
| c26397f1p01230 | 1190 | B9VAS9_SORBI Sucrose synthase OS=Sorghum bicolor GN=SUSY2 PE=3 SV=1 | -8.34 | 6.91 | 4.89E-04 |
| c87962f1p22910 | 2910 | A0A0A7RR05_SACOF Sucrose synthase OS=Saccharum officinarum PE=2 SV=1 | -7.54 | 6.37 | 1.96E-03 |
| c74232f1p0968 | 968 | K7U9J7_MAIZE sucrose-phosphate synthase family OS=Zea mays GN= PE=4 SV=1 | -6.41 | 5.34 | 5.38E-05 |
| c41415f1p01118 | 1126 | Q5U871_9POAL Sucrose transporter OS=Saccharum hybrid cultivar Q117 GN=SUT1 PE=2 SV=1 | -6.16 | 5.16 | 6.80E-03 |
| c42730f1p01426 | 1492 | A5HNE9_SORBI Sucrose non-fermenting related kinase 1b OS=Sorghum bicolor PE=2 SV=1 | -5.98 | 5.03 | 5.26E-03 |
| c105346f1p44268 | 4286 | A5HNE9_SORBI Sucrose non-fermenting related kinase 1b OS=Sorghum bicolor PE=2 SV=1 | -5.62 | 4.72 | 1.00E-02 |
| c106308f1p04384 | 4420 | E1APE3_9POAL Sucrose phosphate synthase A OS=Saccharum hybrid cultivar ROC22 GN=SPSA PE=2 SV=1 | -5.33 | 4.54 | 2.54E-04 |
| c94802f1p53323 | 3324 | E1APE3_9POAL Sucrose phosphate synthase A OS=Saccharum hybrid cultivar ROC22 GN=SPSA PE=2 SV=1 | -5.16 | 4.34 | 8.18E-04 |
| c23771f1p02288 | 2299 | Q5U871_9POAL Sucrose transporter OS=Saccharum hybrid cultivar Q117 GN=SUT1 PE=2 SV=1 | -5.1 | 4.26 | 5.35E-04 |
| c13894f2p1849 | 850 | G3CM26_9POAL Sucrose phosphate synthase OS=Saccharum hybrid cultivar ROC22 PE=2 SV=1 | -4.94 | 4.13 | 2.78E-04 |
| c54637f1p11006 | 1012 | P93783_SACOF Sucrose-phosphate synthase OS=Saccharum officinarum GN= 2 PE=4 SV=1 | -4.8 | 3.98 | 3.15E-05 |
|  |  |  |  |  |  |
| Feature ID | Length (bp) | Description | Fold Change (original values) | Weighted proportions fold change | FDR p-value correction |
| c94324f1p42760 | 2761 | A0A0A1I5C7_9POAL sugar transporter type 2a OS=Saccharum hybrid cultivar R570 PE=3 SV=1 | -12.62 | 10.51 | 9.96E-03 |
| c98146f1p0774 | 817 | Saccharum hybrid cultivar GT28 sugar transporter (ERD6) complete cds | -9.43 | 7.85 | 1.00E-02 |
| c88771f1p01741 | 1704 | C5YNL0_SORBI Bidirectional sugar transporter SWEET OS=Sorghum bicolor GN=Sb08g013620 PE=3 SV=1 | -9.2 | 7.6 | 3.59E-03 |
| c82701f1p01795 | 1777 | C5YNL0_SORBI Bidirectional sugar transporter SWEET OS=Sorghum bicolor GN=Sb08g013620 PE=3 SV=1 | -8.27 | 6.87 | 1.00E-02 |
| c66857f1p11415 | 1416 | B4F8E5_MAIZE Nucleoside-diphosphate-sugar epimerase OS=Zea mays GN=LOC100191908 PE=2 SV=1 | -8.26 | 6.96 | 7.84E-04 |
| c98428f1p03068 | 3092 | A0A0A1I5C7_9POAL sugar transporter type 2a OS=Saccharum hybrid cultivar R570 PE=3 SV=1 | -8.15 | 6.77 | 1.14E-03 |
| c95896f1p03165 | 3073 | Q7X9Y6_9POAL sugar transporter type 2a OS=Saccharum hybrid cultivar Q117 PE=2 SV=1 | -7.61 | 6.37 | 2.91E-03 |
| c86359f1p12077 | 2079 | Q7X9Y6_9POAL sugar transporter type 2a OS=Saccharum hybrid cultivar Q117 PE=2 SV=1 | -7.18 | 6.02 | 4.12E-04 |
| c98548f1p02769 | 2772 | Q7X9Y6_9POAL sugar transporter type 2a OS=Saccharum hybrid cultivar Q117 PE=2 SV=1 | -6.88 | 5.74 | 2.63E-03 |
| c57609f1p01274 | 1271 | O22637_MAIZE SU1 isoamylase OS=Zea mays GN=sugary1 PE=4 SV=1 | -6.05 | 5.03 | 2.07E-03 |
| c55244f1p01935 | 1907 | Q7X9Y6_9POAL sugar transporter type 2a OS=Saccharum hybrid cultivar Q117 PE=2 SV=1 | -5.82 | 4.89 | 2.70E-03 |
| c29857f1p01086 | 1086 | A0A059PZS7_9POAL UDP-sugar pyrophosphorylase OS=Saccharum hybrid cultivar R570 GN=SHCRBa_024_H17_F_300 PE=4 SV=1 | -5.72 | 4.77 | 2.93E-04 |
| c1589f4p31134 | 1134 | C5Y0L1_SORBI Bidirectional sugar transporter SWEET OS=Sorghum bicolor GN=Sb04g012910 PE=3 SV=1 | -4.81 | 4.06 | 4.78E-03 |
| c39872f1p22054 | 2055 | A0A0A6Z9P5_9POAL Sugar transporter OS=Saccharum hybrid cultivar GT28 GN=ERD6 PE=2 SV=1 | -4.78 | 4.01 | 3.27E-03 |
| c30677f1p01777 | 1776 | A0A059PYV8_9POAL Sugar transport OS=Saccharum hybrid cultivar R570 GN=SHCRBa_028_H13_R_100 PE=4 SV=1 | -4.74 | 3.96 | 6.11E-03 |
| c88737f1p02730 | 2740 | A0A0A6Z9P5_9POAL Sugar transporter OS=Saccharum hybrid cultivar GT28 GN=ERD6 PE=2 SV=1 | -4.52 | 3.8 | 9.23E-03 |

**TABLE S18. DEGs obtained in High sugar top vs high sugar bottom with SUGIT database**

| Feature ID | Length (bp) | Description | Fold Change (original values) | Weighted proportions fold change | FDR p-value correction |
| --- | --- | --- | --- | --- | --- |
| c98442f1p02354 | 2367 | Terpene cyclase Sorghum bicolor | -18.71 | 19.23 | 0.00608 |
| c99323f1p0897 | 898 | Putative uncharacterized protein Sorghum bicolor | -15.18 | 15.33 | 0.00457 |
| c18709f1p11993 | 1993 | Putative uncharacterized protein Sorghum bicolor | -10.75 | 10.87 | 0.00608 |
| c3303f3p11218 | 1220 | uncharacterized protein Sb08g018350 Sorghum bicolor | -8.11 | 8.28 | 0.00769 |
| c13506f13p3873 | 874 | Putative uncharacterized protein Sorghum bicolor | -7.77 | 7.97 | 0.0000882 |
| c53655f1p0919 | 919 | Putative uncharacterized protein Sorghum bicolor | -7.64 | 7.63 | 0.00527 |
| c123846f9p61312 | 1313 | Putative uncharacterized protein Sorghum bicolor | -7.59 | 7.64 | 0.00297 |
| c8122f1p0759 | 759 | Uncharacterized protein Zea mays | -7.51 | 7.64 | 0.00658 |
| c61441f1p11782 | 1782 | Phenylalanine ammonia lyase Saccharum hybrid cultivar ROC22 | -6.44 | 6.52 | 0.00382 |
| c54891f1p02222 | 1967 | Putative uncharacterized protein Sorghum bicolor | -6.14 | 6.24 | 0.00608 |
| c115619f2p31644 | 1644 | Phenylalanine ammonia lyase Saccharum hybrid cultivar ROC22 | -5.76 | 5.83 | 0.00394 |
| c57132f1p02223 | 2052 | Putative uncharacterized protein Sorghum bicolor | -5.61 | 5.63 | 0.00047 |
| c33449f1p11192 | 1193 | Putative uncharacterized protein Sorghum bicolor | -5.59 | 5.68 | 0.0000299 |
| c51995f1p1851 | 852 | Putative uncharacterized protein Sorghum bicolor | -5.47 | 5.47 | 0.000167 |
| c71659f1p11192 | 1190 | Putative uncharacterized protein Sorghum bicolor | -5.24 | 5.26 | 0.00623 |
| c11136f1p11199 | 1199 | A0A024DBG9_9POAL FT-like flowering time OS=Saccharum hybrid cultivar GN=FT1 PE=2 SV=1 | -5.17 | 5.34 | 0.00223 |
| c46635f4p1975 | 975 | C5X6A7_SORBI Putative uncharacterized protein Sb02g043510 OS=Sorghum bicolor GN=Sb02g043510 PE=4 SV=1 | -4.18 | 4.17 | 0.00658 |
| c123397f7p51286 | 1288 | C5Y8Y1_SORBI Putative uncharacterized protein Sb06g030260 OS=Sorghum bicolor GN=Sb06g030260 PE=3 SV=1 | -4.05 | 4.13 | 0.0018 |
| c65526f1p02125 | 2132 | C5YEV8_SORBI Putative uncharacterized protein Sb06g014310 OS=Sorghum bicolor GN=Sb06g014310 PE=3 SV=1 | -3.98 | 3.97 | 0.00457 |
| c67448f1p11267 | 1272 | K4PKL8_9POAL LSG OS=Saccharum hybrid cultivar GN=LSG PE=4 SV=1 | -2.54 | 2.59 | 0.00496 |
| c97121f1p23167 | 3230 | C5WQY8_SORBI Putative uncharacterized protein Sb01g041150 OS=Sorghum bicolor GN=Sb01g041150 PE=4 SV=1 | -2.48 | 2.52 | 0.01 |

**TABLE S19. DEGs obtained in low sugar top vs low sugar bottom with SUGIT database**

| Name | Description | Size | Fold Change (original values) | Weighted proportions fold change | FDR p-value correction |
| --- | --- | --- | --- | --- | --- |
| c102953f3p14928 | c102953f3p14928 Callose synthase 12_1.3-beta-D-glucan synthase complex 1. | 4934 | 7.18 | -7.69 | 3.86E-10 |
| c120335f1p15916 | c120335f1p15916 callose synthase 9_1.3-beta-D-glucan synthase complex | 5925 | 3.39 | -3.81 | 1.17E-04 |
| c118560f1p06506 | c118560f1p06506 callose synthase 3-like | 6519 | 5.08 | -5.43 | 1.70E-04 |
| c118656f1p15928 | c118656f1p15928 callose synthase 10_1.3-beta-D-glucan synthase | 5923 | 3.08 | -3.46 | 2.37E-04 |
| c49742f1p0977 | c49742f1p0977 Callose synthase 12_1.3-beta-D-glucan synthase complex Golgi | 977 | -6.09 | 5.1 | 3.50E-04 |
| c39632f1p0732 | c39632f1p0732 callose synthase 9_1.3-beta-D-glucan synthase | 733 | 3.98 | -4.29 | 4.17E-04 |
| c16795f1p01162 | c16795f1p01162 callose synthase 3_1.3-beta-D-glucan synthase | 1162 | -32.61 | 28.07 | 4.17E-04 |
| c117795f1p06197 | c117795f1p06197 callose synthase 10_1.3-beta-D-glucan synthase | 6209 | -8.74 | 7.25 | 4.17E-04 |
| c18520f1p0996 | c18520f1p0996 Retrovirus-related Pol poly LINE-1_1.3-beta-D-glucan synthase complex nuclear pore g 4 iron. 4 | 996 | -10.03 | 8.53 | 4.17E-04 |
| c80857f5p02909 | c80857f5p02909 1.4-alpha-glucan-branching enzyme chloroplastic amyloplastic_1.4-alpha-glucan | 2910 | -5.62 | 4.7 | 4.17E-04 |
| c98137f1p03140 | c98137f1p03140 1.4-alpha-glucan-branching enzyme chloroplastic | 3151 | -7.13 | 6 | 4.17E-04 |
| c51998f1p01203 | c51998f1p01203 probable trehalase_alpha.alpha-trehalase activity | 1204 | -6.05 | 5.1 | 4.17E-04 |
| c41372f1p0959 | c41372f1p0959 sucrose-phosphate synthase_amidophosphoribosyltransferase | 959 | -9.55 | 7.94 | 4.77E-04 |
| c54637f1p11006 | c54637f1p11006 sucrose-phosphate synthase_amidophosphoribosyltransferase | 1012 | -9.28 | 7.73 | 4.77E-04 |
| c45072f1p0861 | c45072f1p0861 sucrose-phosphate synthase_amidophosphoribosyltransferase activity sucrose synthase activity | 914 | -6.85 | 5.67 | 4.77E-04 |
| c13894f2p1849 | c13894f2p1849 sucrose-phosphate synthase_ | 850 | -7.96 | 6.7 | 4.77E-04 |
| c46738f1p23019 | c46738f1p23019 starch branching enzyme I_amyloplast chloroplast 1.4-alpha- sucrose metabolic process | 3022 | -11.86 | 9.86 | 4.77E-04 |
| c98393f1p03043 | c98393f1p03043 1.4-alpha-glucan-branching enzyme chloroplastic amyloplastic-like isoform X1_amyloplast chloroplast 1.4-alpha | 3015 | -7.41 | 6.23 | 4.77E-04 |
| c25733f1p01210 | c25733f1p01210 4-alpha- chloroplastic amyloplastic_amyloplast chloroplast 4-alpha- | 1210 | -8.86 | 7.48 | 4.77E-04 |
| c39076f1p1748 | c39076f1p1748 soluble starch synthase chloroplastic amyloplastic_amyloplast chloroplast stroma | 750 | 5.5 | -5.85 | 4.77E-04 |
| c47269f1p01182 | c47269f1p01182 soluble starch synthase chloroplastic | 1233 | -5.85 | 4.93 | 4.77E-04 |
| c55239f1p11171 | c55239f1p11171 impaired sucrose induction 1_binding | 1183 | -7.36 | 6.12 | 4.77E-04 |
| c23332f3p11222 | c23332f3p11222 impaired sucrose induction 1_binding regulation of carbohydrate metabolic process | 1226 | -8.82 | 7.29 | 5.16E-04 |
| c55061f1p01359 | c55061f1p01359 probable galactinol--sucrose galactosyltransferase 2_catalytic activity | 1359 | -7.58 | 6.31 | 5.16E-04 |
| c54075f1p01398 | c54075f1p01398 probable galactinol--sucrose galactosyltransferase 2_catalytic activity | 1399 | -11.04 | 9.31 | 5.16E-04 |
| c54410f1p01496 | c54410f1p01496 probable galactinol--sucrose galactosyltransferase 2_catalytic activity | 1496 | -4.84 | 4.04 | 5.16E-04 |
| c34273f1p0572 | c34273f1p0572 40S ribosomal S11_cell | 572 | 7.38 | -7.6 | 5.82E-04 |
| c104571f1p04261 | c104571f1p04261 nodal modulator 1_cell wall endomembrane system membrane | 4430 | -9.41 | 7.95 | 5.82E-04 |
| c42342f1p0769 | c42342f1p0769 conserved oligomeric Golgi complex subunit 4_ | 772 | -7.27 | 6.13 | 6.25E-04 |
| c14100f2p41600 | c14100f2p41600 histidinol chloroplastic isoform X1_cell wall mitochondrion chloroplast stroma | 1602 | 5.72 | -6.08 | 6.25E-04 |
| c46996f1p01335 | c46996f1p01335 alanine aminotransferase 2_cell wall mitochondrion integral component of | 1335 | -8.27 | 6.95 | 7.87E-04 |
| c47176f3p01209 | c47176f3p01209 pectinesterase 31_cell wall mitochondrion negative bacterium starch metabolic process sucrose metabolic process | 1211 | -4.24 | 3.54 | 7.87E-04 |
| c9373f1p21337 | c9373f1p21337 neutral alkaline invertase chloroplastic-like_chloroplast glucosidase II complex sucrose alpha-glucosidase activity | 1338 | 4.52 | -4.73 | 7.87E-04 |
| c85234f1p03420 | c85234f1p03420 NHL repeat-containing 2_chloroplast thylakoid chloroplast stroma integral metabolic process | 3431 | -5.48 | 4.62 | 7.87E-04 |
| c1132f3p01641 | c1132f3p01641 4-diphosphocytidyl-2-C-methyl-D-erythritol | 1644 | 7.99 | -8.4 | 8.01E-04 |
| c33787f1p11661 | c33787f1p11661 sucrose transport SUC4-like_cis-Golgi network integral transmembrane transport | 1676 | -5.43 | 4.54 | 8.39E-04 |
| c27509f6p11691 | c27509f6p11691 UDP-D-apiose UDP-D-xylose synthase 2_cytoplasm dTDP-glucose 4.6-dehydratase activity UDP-glucuronate | 1689 | -5.82 | 4.94 | 8.39E-04 |
| c86378f1p23317 | c86378f1p23317 UTP--glucose-1-phosphate uridylyltransferase_cytoplasm UTP:glucose-1-phosphate | 3321 | -5.17 | 4.3 | 8.50E-04 |
| c17115f1p11050 | c17115f1p11050 UTP--glucose-1-phosphate uridylyltransferase_cytoplasm UTP: | 1050 | -5.42 | 4.46 | 8.57E-04 |
| c73413f1p11483 | c73413f1p11483 UTP--glucose-1-phosphate uridylyltransferase_cytoplasm UTP:glucose-1-phosphate uridylyltransferase | 1483 | -4.83 | 4.04 | 8.57E-04 |
| c28448f1p01331 | c28448f1p01331 probable fructokinase-1_cytoplasmic. metabolic process | 1327 | -12.85 | 10.97 | 8.57E-04 |
| c43087f1p0992 | c43087f1p0992 ADP-glucose pyrophosphorylase small subunit_cytosol amyloplast chloroplast stroma heterotetrameric ADPG pyrophosphorylase complex apoplast | 1050 | -6.65 | 5.66 | 8.57E-04 |
| c12256f3p21988 | c12256f3p21988 glucose-1-phosphate adenylyltransferase small chloroplastic amyloplastic_cytosol | 1990 | -10.2 | 8.47 | 9.11E-04 |
| c33012f1p0860 | c33012f1p0860 AF287482_5 partial_cytosol chloroplast envelope integral component of membrane | 863 | -9.76 | 8.08 | 9.47E-04 |
| c62044f1p0942 | c62044f1p0942 probable fructokinase- chloroplastic_cytosol chloroplast stroma ribokinase | 945 | -24.01 | 19.92 | 9.47E-04 |
| c22688f3p81998 | c22688f3p81998 cytosolic phosphoglucose partial_cytosol | 1999 | 5.32 | -5.68 | 9.47E-04 |
| c64397f1p01933 | c64397f1p01933 cytosolic phosphoglucose partial_cytosol glucose-6-phosphate isomerase activity gluconeogenesis glycolytic process response to sucrose flavonoid biosynthetic process | 2008 | -7.22 | 5.97 | 1.00E-03 |
| c45366f1p12001 | c45366f1p12001 probable alpha.alpha-trehalose-phosphate synthase [ | 2061 | -8.4 | 7 | 1.00E-03 |
| c29857f1p01086 | c29857f1p01086 UDP-sugar pyrophosphorylase_cytosol pollen tube UTP:glucose-1-phosphate | 1086 | -4.42 | 3.7 | 1.00E-03 |
| c15474f1p2553 | c15474f1p2553 probable alkaline neutral invertase D_cytosol sucrose alpha-glucosidase activity glycopeptide alpha-N- | 553 | -7.35 | 6.13 | 1.00E-03 |
| c37461f1p11221 | c37461f1p11221 serine threonine- kinase CTR1_endoplasmic reticulum membrane integral component of membrane MAP kinase kinase kinase | 1221 | -8.36 | 6.96 | 1.01E-03 |
| c27155f1p02291 | c27155f1p02291 cellulose synthase A catalytic subunit 3 [UDP-forming]_endosome trans-Golgi network plasma membrane plasmodesma integral component ofprocess | 2337 | -6.28 | 5.21 | 1.03E-03 |
| c40661f1p01222 | c40661f1p01222 rhodanese-like domain-containing chloroplastic_extracellular region | 1229 | -24.65 | 21.05 | 1.03E-03 |
| c14115f1p01176 | c14115f1p01176 rhodanese-like domain-containing chloroplastic_extracellular region electron transport in photosystem II photosynthetic electron transport in | 1176 | -5.34 | 4.52 | 1.03E-03 |
| c23556f4p41863 | c23556f4p41863 beta-glucosidase 44-like_extracellular region mitochondrion beta-galactosidase | 1863 | -7.63 | 6.45 | 1.03E-03 |
| c48479f1p0692 | c48479f1p0692 probable polygalacturonase_extracellular region mitochondrion integral component of membrane polygalacturonase activity lyase activity cell wall organization starch metabolic process sucrose metabolic process | 694 | 3.77 | -4.04 | 1.03E-03 |
| c2458f1p01602 | c2458f1p01602 translation factor GUF1 mitochondrial_extracellular region nucleus mitochondrial inner membrane mitochondrial matrix DNA | 1607 | 3.8 | -4.03 | 1.03E-03 |
| c97007f1p21604 | c97007f1p21604 probable alpha-amylase 2_extracellular region nucleus nucleic acid binding alpha-amylase activity calcium ion binding phosphoric diester hydrolase activity zinc ion | 1607 | -8.34 | 7.08 | 1.03E-03 |
| c24251f1p01167 | c24251f1p01167 probable polygalacturonase_extracellular region plastid integral component of membrane polygalacturonase activity | 1181 | -5.68 | 4.74 | 1.03E-03 |
| c9922f1p01537 | c9922f1p01537 probable polygalacturonase_extracellular region vacuolar membrane | 1552 | -5.19 | 4.31 | 1.03E-03 |
| c25717f1p21255 | c25717f1p21255 probable polygalacturonase_extracellular region vacuole integral component of membrane cytoplasmic. | 1255 | -7.03 | 5.88 | 1.03E-03 |
| c42155f1p01433 | c42155f1p01433 alpha-amylase partial_extracellular space integral component of membrane cytoplasmic. | 1435 | -32.19 | 26.99 | 1.03E-03 |
| c12868f1p1732 | c12868f1p1732 receptor homology transmembrane domain- and RING domain-containing 1_extrinsic component into peroxisome matrix | 732 | -9.62 | 8.05 | 1.10E-03 |
| c98557f1p02355 | c98557f1p02355 probable galactinol--sucrose galactosyltransferase 1_galactinol-raffinose galactose metabolic process | 2360 | -7.11 | 6 | 1.10E-03 |
| c87662f1p02298 | c87662f1p02298 glucose-6-phosphate isomerase chloroplastic_glucose-6-phosphate isomerase activity gluconeogenesis glycolytic pentose-phosphate shunt | 2304 | -7.22 | 6.1 | 1.16E-03 |
| c83216f1p23207 | c83216f1p23207 probable starch synthase chloroplastic amyloplastic_glycogen (starch) synthase activity starch metabolic process sucrose metabolic process | 3216 | -7.07 | 5.9 | 1.16E-03 |
| c10824f1p0909 | c10824f1p0909 sucrose transport SUC3 isoform X2_Golgi apparatus integral transmembrane transport | 912 | 4.73 | -4.96 | 1.16E-03 |
| c13251f2p0549 | c13251f2p0549 probable beta-1.4-xylosyltransferase IRX10L_Golgi membrane biogenesis glucuronoxylan biosynthetic process cell wall organization starch metabolic process sucrose metabolic process nucleotide metabolic process | 550 | -10.71 | 8.85 | 1.24E-03 |
| c29741f1p0857 | c29741f1p0857 UDP-glucuronic acid decarboxylase 2-like_Golgi membrane endosome trans-Golgi process | 860 | -5.3 | 4.4 | 1.28E-03 |
| c49104f1p02396 | c49104f1p02396 probable galacturonosyltransferase 11_Golgi membrane integral component of wall organization | 2392 | -4.05 | 3.41 | 1.31E-03 |
| c68959f1p01972 | c68959f1p01972 probable galacturonosyltransferase 10_Golgi membrane mitochondrion endosome trans-Golgi network integral component of membrane organization | 1974 | -5.77 | 4.87 | 1.31E-03 |
| c98268f1p02640 | c98268f1p02640 Polygalacturonate 4-alpha-galacturonosyltransferase_Golgi membrane mitochondrion endosome trans-Golgi network integral sucrose metabolic process nucleotide metabolic process | 2637 | -4.24 | 3.59 | 1.31E-03 |
| c58658f1p02169 | c58658f1p02169 probable galacturonosyltransferase 3 isoform X1_Golgi membrane mitochondrion endosome vacuolar membrane process | 2283 | -4.98 | 4.13 | 1.31E-03 |
| c53203f1p01822 | c53203f1p01822 glucan endo-1.3-beta-glucosidase 5_integral component of membrane cytoplasmic. membrane-bounded process sucrose metabolic process | 1822 | -7.47 | 6.13 | 1.31E-03 |
| c56409f1p0594 | c56409f1p0594 probable fructokinase-1_integral component of membrane cytoplasmic. metabolic process | 596 | 3.95 | -4.16 | 1.31E-03 |
| c23771f1p02288 | c23771f1p02288 sucrose transporter 1_integral component of plasma membrane maltose:proton symporter activity sucrose:proton transport | 2299 | 3.9 | -4.1 | 1.31E-03 |
| c62911f1p0883 | c62911f1p0883 sucrose transport SUC3 isoform X2_integral component of plasma membrane maltose:proton symporter activity sucrose: salicin transport transmembrane transport cation transport | 893 | -6.29 | 5.34 | 1.31E-03 |
| c16576f1p0671 | c16576f1p0671 Sucrose Transporter_integral component of plasma membrane maltose:proton symporter activity sucrose:proton symporter activity salicin transmembrane transporter activity sucrose metabolic process pollen germination maltose transport sucrose transport salicin transport transmembrane transport cation transport | 671 | -4.71 | 3.91 | 1.31E-03 |
| c30737f2p01022 | c30737f2p01022 sucrose transport SUC3 isoform X2_integral component of plasma membrane maltose:proton symporter activity sucrose:proton symporter activity salicin transmembrane transporter transport cation transport | 1023 | -5.09 | 4.33 | 1.31E-03 |
| c41415f1p01118 | c41415f1p01118 sucrose transport SUC3 isoform X2_integral component of plasma membrane maltose:proton symporter activity sucrose:proton symporter activity salicin cation transport | 1126 | -6.02 | 4.97 | 1.31E-03 |
| c65976f2p01948 | c65976f2p01948 sucrose transport SUC4-like_integral component of plasma membrane sucrose transmembrane transporter activity sucrose transport transmembrane transport | 1948 | -56.64 | 46.92 | 1.31E-03 |
| c74336f3p11944 | c74336f3p11944 sucrose transport SUC4-like_integral component of plasma membrane sucrose transmembrane transporter activity sucrose transport transmembrane transport | 1951 | -6.17 | 5.21 | 1.31E-03 |
| c11531f1p0867 | c11531f1p0867 sucrose transport SUC3 isoform X1_integral component of plasma membrane sucrose transmembrane transporter activity symporter activity sucrose metabolic process sucrose transport transmembrane transport | 867 | -7.64 | 6.46 | 1.31E-03 |
| c63072f1p21490 | c63072f1p21490 cellulose synthase A catalytic subunit 3 [UDP-forming]_membrane cellulose synthase (UDP-forming) activity cellulose biosynthetic process starch metabolic process sucrose metabolic process UDP-glucose metabolic process | 1490 | -5.33 | 4.46 | 1.31E-03 |
| c114283f1p43649 | c114283f1p43649 cellulose synthase A catalytic subunit 3 [UDP-forming]_membrane cellulose synthase (UDP-forming) activity cellulose biosynthetic process starch metabolic process sucrose metabolic process UDP-glucose metabolic process | 3657 | -15.95 | 13.23 | 1.31E-03 |
| c91177f5p63634 | c91177f5p63634 cellulose synthase A catalytic subunit 3 [UDP-forming]_membrane cellulose synthase (UDP-forming) activity process | 3636 | -6.95 | 5.91 | 1.31E-03 |
| c2358f1p0607 | c2358f1p0607 transmembrane 53-like_membrane intracellular membrane-bounded organelle process sucrose metabolic process UDP-glucose metabolic process | 607 | -6.88 | 5.69 | 1.31E-03 |
| c87054f1p02277 | c87054f1p02277 hexokinase-1-like_mitochondrial inner membrane cytosol chloroplast outer membrane phosphate metabolic process | 2188 | -5.87 | 4.93 | 1.31E-03 |
| c16980f1p1486 | c16980f1p1486 isovaleryl- mitochondrial_mitochondrial matrix integral component of membrane metabolic process | 486 | -7.71 | 6.36 | 1.31E-03 |
| c48226f1p01342 | c48226f1p01342 alkaline neutral invertase mitochondrial_mitochondrion chloroplast sucrose alpha- | 1344 | -7.48 | 6.25 | 1.31E-03 |
| c47752f1p11619 | c47752f1p11619 homogentisate phytyltransferase chloroplastic isoform X1_mitochondrion chloroplast thylakoid membrane organization | 1636 | -11.14 | 9.29 | 1.31E-03 |
| c42815f1p0983 | c42815f1p0983 hexokinase-1-like_mitochondrion cytosol chloroplast outer membrane integral component of membrane | 984 | -5.37 | 4.56 | 1.31E-03 |
| c44590f1p11025 | c44590f1p11025 Hexokinase-1_mitochondrion cytosol chloroplast outer membrane integral component of membrane glucokinase activity ATP binding glucose binding fructokinase activity mannokinase activity cellular | 1018 | -7.13 | 6.01 | 1.31E-03 |
| c87456f1p02231 | c87456f1p02231 hexokinase-1_mitochondrion cytosol chloroplast outer membrane integral component of membrane glucokinase activity ATP binding e metabolic process | 2054 | -8.78 | 7.29 | 1.31E-03 |
| c54421f1p1932 | c54421f1p1932 hexokinase-1_mitochondrion cytosol chloroplast outer membrane integral component of membrane glucokinase activity ATP binding | 936 | -51.75 | 42.81 | 1.31E-03 |
| c35595f1p11176 | c35595f1p11176 sucrose nonfermenting 4_mitochondrion integral component of membrane | 1176 | 4.29 | -4.49 | 1.31E-03 |
| c43095f1p0751 | c43095f1p0751 alpha.alpha- process sucrose metabolic process | 751 | -6.62 | 5.49 | 1.31E-03 |
| c31343f1p01300 | c31343f1p01300 glucan endo-1.3-beta-glucosidase 14_mitochondrion integral component of membrane anchored component of plasma glucosidase activity starch metabolic process sucrose metabolic process | 1301 | -16.65 | 13.94 | 1.31E-03 |
| c58652f1p01952 | c58652f1p01952 lactation elevated 1_mitochondrion integral component of membrane ATP | 1952 | -5.67 | 4.71 | 1.37E-03 |
| c26397f1p01230 | c26397f1p01230 sucrose synthase 1_mitochondrion integral component of membrane sucrose synthase activity sucrose metabolic process biosynthetic process response to carbon dioxide starch metabolic process | 1190 | -5 | 4.16 | 1.37E-03 |
| c23473f12p11676 | c23473f12p11676 UDP-glucuronic acid decarboxylase 1-like_ | 1676 | -7.5 | 6.27 | 1.48E-03 |
| c25634f4p11826 | c25634f4p11826 O-linked-mannose beta-1.4-N-acetylglucosaminyltransferase 2-like_ process | 1827 | -5.64 | 4.83 | 1.48E-03 |
| c3055f1p01040 | c3055f1p01040 Histidine kinase 3_mitochondrion plasma membrane negative regulation of iron ion | 1040 | -5.26 | 4.41 | 1.48E-03 |
| c77338f1p01213 | c77338f1p01213 chlorophyll a-b binding chloroplastic_mitochondrion PSII associated light-harvesting | 1218 | -7.32 | 6.17 | 1.48E-03 |
| c20933f1p0495 | c20933f1p0495 dihydroflavonol-4-reductase_mitochondrion vacuolar membrane endoplasmic reticulum plasma membrane dihydrokaempferol 4-reductase | 497 | -5.09 | 4.22 | 1.48E-03 |
| c91956f1p12882 | c91956f1p12882 origin of replication complex subunit 3_nuclear origin of replication recognition complex plastid DNA binding DNA replication response to sucrose lateral root development | 2888 | 4.03 | -4.24 | 1.52E-03 |
| c56741f1p01312 | c56741f1p01312 fructose-1.6- cytosolic_nucleus cytosol integral component of membrane 2-alkenal reductase [NAD(P)] activity fructose 1.6-bisphosphate 1-phosphatase activity metal ion binding starch catabolic process sucrose | 1312 | -10.7 | 8.96 | 1.52E-03 |
| c67490f1p61598 | c67490f1p61598 fructose-1.6- cytosolic_nucleus cytosol integral phosphate shunt carbon utilization | 1600 | -5.03 | 4.25 | 1.52E-03 |
| c36760f1p0434 | c36760f1p0434 26S proteasome non-ATPase regulatory subunit 4 | 437 | -9.91 | 8.34 | 1.52E-03 |
| c88334f1p03322 | c88334f1p03322 transcription elongation factor SPT5 homolog 1 | 3312 | -10 | 8.4 | 1.53E-03 |
| c14863f1p01380 | c14863f1p01380 beta-amylase chloroplastic_nucleus transcription | 1386 | -7.86 | 6.6 | 1.53E-03 |
| c22664f1p0724 | c22664f1p0724 probable sucrose-phosphate synthase 1_plasma | 724 | -5.86 | 4.85 | 1.55E-03 |
| c1480f7p1788 | c1480f7p1788 AP-2 complex subunit sigma_plasma membrane clathrin- | 788 | -5.36 | 4.47 | 1.56E-03 |
| c18002f4p0849 | c18002f4p0849 AP-2 complex subunit sigma_plasma membrane clathrin-coated pit integral | 849 | -12.54 | 10.43 | 1.56E-03 |
| c87720f1p02485 | c87720f1p02485 probable cellulose synthase A catalytic subunit 6 [UDP-forming] isoform X1_plasma | 2488 | -11.48 | 9.6 | 1.56E-03 |
| c83778f1p21660 | c83778f1p21660 serine arginine-rich SC35-like splicing factor | 1677 | -9.95 | 8.37 | 1.56E-03 |
| c61414f1p01349 | c61414f1p01349 probable polygalacturonase_polygalacturonase activity starch metabolic process sucrose metabolic process | 1352 | 3.81 | -4.02 | 1.56E-03 |
| c120440f1p22050 | c120440f1p22050 probable polygalacturonase_polygalacturonase activity starch metabolic process sucrose metabolic process | 2053 | -8.41 | 7.09 | 1.56E-03 |
| c95656f1p0855 | c95656f1p0855 galacturonosyltransferase 8_polygalacturonate 4-alpha-galacturonosyltransferase activity starch metabolic process sucrose metabolic process nucleotide metabolic process | 857 | -10.93 | 9.23 | 1.56E-03 |
| c49849f4p11254 | c49849f4p11254 Autophagy-related 101_pre-autophagosomal structure cellulase activity autophagosome assembly cellulose catabolic process starch metabolic process sucrose metabolic process | 1254 | -10.14 | 8.33 | 1.56E-03 |
| c1460f4p01125 | c1460f4p01125 Autophagy-related 101_pre-autophagosomal structure cellulase activity autophagosome assembly starch metabolic process sucrose metabolic process cellulose catabolic process | 1127 | 3.81 | -4.01 | 1.56E-03 |
| c106308f1p04384 | c106308f1p04384_probable sucrose-phosphate synthase 1 | 4420 | -5.6 | 4.64 | 1.56E-03 |
| c111075f1p04588 | c111075f1p04588 sucrose synthase_sucrose synthase activity sucrose metabolic process starch metabolic process | 4585 | -5.31 | 4.41 | 1.56E-03 |
| c113428f1p14461 | c113428f1p14461 probable sucrose-phosphate synthase 1_sucrose synthase activity sucrose-phosphate synthase activity sucrose metabolic process starch metabolic process | 4465 | -9.98 | 8.39 | 1.56E-03 |
| c94802f1p53323 | c94802f1p53323 probable sucrose-phosphate synthase 1_sucrose synthase activity sucrose-phosphate synthase activity sucrose metabolic process starch metabolic process | 3324 | -6.51 | 5.44 | 1.56E-03 |
| c114443f1p04580 | c114443f1p04580_sucrose transport SUC3 isoform X2 | 4680 | -4.02 | 3.39 | 1.56E-03 |
| c111151f1p124446 | c111151f1p124446 UDP-glucose 6-dehydrogenase 4_UDP-glucose 6-dehydrogenase activity NAD binding process | 4467 | -7.15 | 6.08 | 1.56E-03 |
| c67348f1p0916 | c67348f1p0916 carbohydrate-binding-like fold partial_vacuolar membrane endoplasmic reticulum Golgi apparatus plant-type cell wall | 940 | -8.67 | 7.29 | 1.56E-03 |
| c26192f1p01764 | c26192f1p01764 glucan endo-1.3-beta-glucosidase 6_vacuole plant-type cell wall plasmodesma integral component of membrane | 1768 | -6.28 | 5.17 | 1.56E-03 |
| c27509f6p11691 | c27509f6p11691 UDP-D-apiose UDP-D-xylose synthase 2_cytoplasm dTDP-glucose 4.6-dehydratase activity UDP-glucuronate | 1689 | -5.44 | 4.49 | 1.56E-03 |
| c41307f1p0705 | c41307f1p0705 eukaryotic translation initiation factor 3 subunit E_cytosol eukaryotic translation initiation factor 3 complex plasma flower development meristem | 705 | -6.19 | 5.11 | 1.56E-03 |
| c17031f7p11245 | c17031f7p11245 haloacid dehalogenase-like hydrolase domain-containing Sgpp_cytosol | 1246 | -5.73 | 4.74 | 1.56E-03 |
| c29857f1p01086 | c29857f1p01086 UDP-sugar pyrophosphorylase_cytosol pollen tube UTP:glucose-1-phosphate uridylyltransferase activity UTP:arabinose-1-phosphate | 1086 | -7.82 | 6.59 | 1.56E-03 |
| c15101f4p21116 | c15101f4p21116 Xanthoxin dehydrogenase_cytosol xanthoxin dehydrogenase activity proline biosynthetic process response to heat response to water deprivation response to fructose regulation of abscisic acid biosynthetic process sugar mediated signaling pathway oxidation-reduction process | 1117 | -8.21 | 6.86 | 1.56E-03 |
| c97033f1p02667 | c97033f1p02667 Trifunctional UDP-glucose 4.6-dehydratase UDP-4-keto-6-deoxy-D-glucose 3.5- | 2670 | -16.18 | 13.73 | 1.56E-03 |
| c37461f1p11221 | c37461f1p11221 serine threonine- kinase CTR1_endoplasmic reticulum membrane integral component of membrane MAP kinase kinase | 1221 | -6.92 | 5.88 | 1.56E-03 |
| c17619f4p0788 | c17619f4p0788 cytochrome b5_endoplasmic reticulum membrane integral component of complex | 791 | -10.2 | 8.63 | 1.56E-03 |
| c17809f3p2662 | c17809f3p2662 cytochrome b5_endoplasmic reticulum membrane integral component of complex | 665 | -6.61 | 5.47 | 1.56E-03 |
| c34693f2p2690 | c34693f2p2690 cytochrome b5_endoplasmic reticulum membrane integral component of membrane organelle membrane complex | 692 | -6.13 | 5.06 | 1.56E-03 |
| c883f6p11232 | c883f6p11232 NADH--cytochrome b5 reductase 1_endoplasmic reticulum plasma membrane plant-type cell wall integral component of | 1231 | -8.81 | 7.28 | 1.56E-03 |
| c11650f1p0822 | c11650f1p0822 NADH--cytochrome b5 reductase 1_endoplasmic electron transport chain | 822 | -5.78 | 4.78 | 1.56E-03 |
| c56847f1p12479 | c56847f1p12479 glutamine--fructose-6-phosphate aminotransferase [isomerizing] 2-like_ biosynthetic process | 2481 | -11.82 | 9.86 | 1.56E-03 |
| c38473f1p41068 | c38473f1p41068 probable sugar phosphate phosphate translocator At3g11320_Golgi apparatus integral component of membrane carbohydrate transport | 1069 | -8.92 | 7.51 | 1.56E-03 |
| c86059f1p11715 | c86059f1p11715 GDP-mannose transporter GONST3-like_Golgi apparatus integral component of membrane carbohydrate transport nucleotide-sugar transport | 1714 | -6.67 | 5.55 | 1.56E-03 |
| c37944f2p01763 | c37944f2p01763 UDP-galactose transporter 1_Golgi apparatus integral component of membrane phosphorylation | 1766 | 4.21 | -4.47 | 1.56E-03 |
| c50090f2p11512 | c50090f2p11512 UDP-galactose transporter 1_Golgi apparatus integral component of membrane cyclin-dependent protein serine/threonine kinase activity carbohydrate transport nucleotide- | 1522 | -5.74 | 4.77 | 1.56E-03 |
| c56025f1p11224 | c56025f1p11224 GDP-mannose transporter GONST4_Golgi apparatus integral component of membrane cytoplasmic. membrane-bounded vesicle carbohydrate transport nucleotide-sugar transport | 1240 | -6.24 | 5.16 | 1.56E-03 |
| c29741f1p0857 | c29741f1p0857 UDP-glucuronic acid decarboxylase 2-like_Golgi membrane endosome trans-Golgi metabolic process | 860 | -6.09 | 5.05 | 1.59E-03 |
| c95279f1p11869 | c95279f1p11869 CMP-sialic acid transporter 3-like_Golgi membrane integral component of membrane sugar:proton symporter activity carbohydrate transport | 1872 | -5.55 | 4.6 | 1.59E-03 |
| c52017f1p11861 | c52017f1p11861 CMP-sialic acid transporter 3-like_Golgi membrane integral component of membrane sugar:proton symporter activity carbohydrate transport | 1879 | -7.85 | 6.59 | 1.60E-03 |
| c55840f1p01629 | c55840f1p01629 CMP-sialic acid transporter 1_Golgi membrane integral component of membrane sugar: | 1627 | -11.87 | 9.89 | 1.61E-03 |
| c16820f1p11181 | c16820f1p11181 CMP-sialic acid transporter 1_Golgi membrane integral component of membrane | 1181 | -7 | 5.9 | 1.62E-03 |
| c83106f1p01601 | c83106f1p01601 bidirectional sugar transporter SWEET1-like_integral component of membrane | 1572 | -7.31 | 6.13 | 1.62E-03 |
| c82205f1p31160 | c82205f1p31160 bidirectional sugar transporter SWEET4-like_integral component of membrane | 1160 | -6.52 | 5.49 | 1.65E-03 |
| c74533f1p01696 | c74533f1p01696 probable sugar phosphate phosphate translocator At5g25400_integral component of membrane cytoplasmic. membrane-bounded vesicle carbohydrate transport | 1699 | -5.59 | 4.69 | 1.65E-03 |
| c17652f5p01677 | c17652f5p01677 UDP-galactose transporter 2-like_integral component of membrane nucleotide-sugar transmembrane transporter activity carbohydrate transport nucleotide-sugar transport nucleotide transmembrane transport | 1681 | -7.34 | 6.14 | 1.67E-03 |
| c73460f1p01624 | c73460f1p01624 sugar transporter ERD6-like 16_integral component of membrane substrate-specific transmembrane transporter activity transmembrane transport | 1624 | -5.28 | 4.41 | 1.68E-03 |
| c55794f2p21702 | c55794f2p21702 sugar transporter ERD6-like 5_integral component of membrane substrate-specific transmembrane transporter activity transmembrane transport | 1703 | -6.44 | 5.34 | 1.69E-03 |
| c33072f3p11993 | c33072f3p11993 plastidic glucose transporter 4_integral component of | 1995 | -5.18 | 4.31 | 1.70E-03 |
| c85200f1p22742 | c85200f1p22742 monosaccharide-sensing 2-like_integral component of plasma membrane plant-type | 2745 | -14.43 | 12.31 | 1.71E-03 |
| c96752f1p02674 | c96752f1p02674 monosaccharide-sensing 2-like_integral component of plasma membrane plant-type | 2648 | -10.71 | 9.12 | 1.73E-03 |
| c94982f1p02826 | c94982f1p02826 monosaccharide-sensing 2_integral component of glucose transmembrane transport | 2809 | -9 | 7.56 | 1.75E-03 |
| c97216f1p03269 | c97216f1p03269 monosaccharide-sensing 2-like_integral component of plasma membrane plant-type transmembrane transport | 2818 | -8.55 | 7.2 | 1.75E-03 |
| c119430f1p02798 | c119430f1p02798 monosaccharide-sensing 2-like_integral component of plasma membrane plant-type glucose transmembrane transport | 2692 | -5.98 | 5.02 | 1.77E-03 |
| c43902f1p0933 | c43902f1p0933 bidirectional sugar transporter SWEET3_integral | 933 | -6.65 | 5.51 | 1.79E-03 |
| c1589f4p31134 | c1589f4p31134 bidirectional sugar transporter SWEET4-like_integral component of plasma membrane sugar transmembrane transporter activity carbohydrate transmembrane transport | 1134 | -5.65 | 4.76 | 1.79E-03 |
| c39872f1p22054 | c39872f1p22054 Sugar transporter ERD6-like 6_integral component of plasma membrane sugar:proton | 2055 | -8.61 | 7.08 | 1.79E-03 |
| c57636f1p12053 | c57636f1p12053 probable polyol transporter 4_integral component of plasma membrane sugar:proton transmembrane transport | 2064 | -6.62 | 5.56 | 1.79E-03 |
| c88737f1p02730 | c88737f1p02730 Sugar transporter ERD6-like 6_integral component of plasma membrane sugar:proton oxidation-reduction process glucose transmembrane transport | 2740 | -6 | 5.05 | 1.79E-03 |
| c28431f1p12020 | c28431f1p12020 Sugar transporter ERD6-like 6_integral component of plasma membrane sugar:proton symporter activity glucose transmembrane transport | 2025 | -7.72 | 6.48 | 1.79E-03 |
| c31441f1p41927 | c31441f1p41927 D-xylose-proton symporter-like 2_integral component of plasma membrane | 1930 | -5.22 | 4.32 | 1.79E-03 |
| c52810f1p0952 | c52810f1p0952 sugar transporter ERD6-like 5_integral component of | 988 | -7.66 | 6.44 | 1.79E-03 |
| c44668f2p11655 | c44668f2p11655 sugar transport 5_integral component of plasma | 1664 | 3.85 | -4.06 | 1.79E-03 |
| c57562f1p01268 | c57562f1p01268 NADH-cytochrome b5 reductase_mitochondrial intermembrane space Golgi | 1277 | -12.3 | 10.3 | 1.79E-03 |
| c104083f1p04536 | c104083f1p04536 ABC transporter C family member 15_mitochondrion endoplasmic reticulum membrane process | 4545 | -9.46 | 7.91 | 1.79E-03 |
| c112102f1p04847 | c112102f1p04847 retrotransposon unclassified_mitochondrion | 4837 | -5.87 | 4.93 | 1.79E-03 |
| c69285f1p03803 | c69285f1p03803 monosaccharide- | 3691 | -5.35 | 4.51 | 1.79E-03 |
| c114048f1p04553 | c114048f1p04553 retrotransposon unclassified_mitochondrion plastid system DNA integration | 4568 | -5.8 | 4.77 | 1.79E-03 |
| c10888f1p0835 | c10888f1p0835 ubiquitin carboxyl-terminal hydrolase 26_nucleolus thiol-dependent ubiquitin-specific | 836 | -9.05 | 7.53 | 1.79E-03 |
| c86030f1p02320 | c86030f1p02320 SNF1-related kinase catalytic subunit alpha KIN10_nucleus cytoplasm cAMP- | 2357 | -7.39 | 6.11 | 1.79E-03 |
| c11352f1p51622 | c11352f1p51622 SNF1-related kinase catalytic subunit alpha | 1632 | -5.27 | 4.38 | 1.79E-03 |
| c30296f2p11991 | c30296f2p11991 SNF1-related kinase catalytic subunit alpha KIN10_nucleus cytoplasm cAMP- | 1992 | -9.29 | 7.64 | 1.79E-03 |
| c10863f1p01152 | c10863f1p01152 nuclear cap-binding subunit 1_nucleus histone H3-K9 methylation | 1128 | -4.63 | 3.87 | 1.79E-03 |
| c42187f1p11882 | c42187f1p11882 SNF1-related kinase catalytic subunit alpha KIN10_nucleus cytoplasm protein family amino acid metabolic process | 1902 | -10.6 | 8.98 | 1.79E-03 |
| c48950f1p01701 | c48950f1p01701 arginine N-methyltransferase_nucleus cytosol integral component of membrane - | 1716 | -12.35 | 10.19 | 1.79E-03 |
| c66218f1p01848 | c66218f1p01848 arginine N-methyltransferase_nucleus cytosol integral component of membrane ATPase activator activity histone- response to freezing | 1867 | -6.06 | 5.11 | 1.79E-03 |
| c52008f1p0654 | c52008f1p0654 cullin-4_nucleus cytosol plastid Cul4-RING E3 ubiquitin ligase complex ligase activity ubiquitin protein ligase se to freezing cell division | 715 | -6.68 | 5.58 | 1.79E-03 |
| c81760f1p03027 | c81760f1p03027 cullin-4_nucleus cotyledon development response to freezing cell division | 3036 | -8.41 | 7.05 | 1.79E-03 |
| c15183f1p01127 | c15183f1p01127 cullin-4_nucleus cytosol plastid Cul4-RING E3 ubiquitin ligase complex ligase activity ubiquitin protein ligase freezing cell division | 1128 | -8.51 | 7.11 | 1.79E-03 |
| c13896f2p01778 | c13896f2p01778 peroxisomal membrane PEX14 isoform X1_ biosynthetic process | 1778 | -4.56 | 3.84 | 1.79E-03 |
| c95529f1p01780 | c95529f1p01780 peroxisomal membrane PEX14 isoform X1_peroxisomal membrane integral biosynthetic process | 1803 | -5.45 | 4.51 | 1.79E-03 |
| c18543f1p1760 | c18543f1p1760 serine threonine- kinase ACR4_plasma membrane cell surface integral component of serine family amino acid metabolic process | 760 | -4.99 | 4.14 | 1.82E-03 |
| c1480f7p1788 | c1480f7p1788 AP-2 complex subunit sigma_plasma membrane clathrin-coated pit integral component of membrane membrane coat | 788 | -10.36 | 8.63 | 1.83E-03 |
| c18002f4p0849 | c18002f4p0849 AP-2 complex subunit sigma_plasma membrane clathrin-coated pit integral | 849 | -5.97 | 4.92 | 1.85E-03 |
| c91102f2p12010 | c91102f2p12010_probable sugar phosphate phosphate translocator At1g06470 | 2015 | 3.97 | -4.17 | 1.86E-03 |
| c76422f1p01501 | c76422f1p01501_probable sugar phosphate phosphate translocator At3g14410 | 1502 | -8.26 | 7.04 | 1.86E-03 |
| c86858f1p0687 | c86858f1p0687_probable sugar phosphate phosphate translocator At5g25400 | 687 | -5.14 | 4.26 | 1.87E-03 |
| c47862f1p11961 | c47862f1p11961 UDP-arabinose 4-epimerase 1_UDP-glucose 4-epimerase activity galactose metabolic process nucleotide metabolic process nucleotide-sugar metabolic process | 1965 | -5.62 | 4.67 | 1.87E-03 |
| c51862f1p01658 | c51862f1p01658 UDP-arabinose 4-epimerase 1_UDP-glucose 4-epimerase activity galactose metabolic process nucleotide metabolic process nucleotide-sugar metabolic process | 1648 | -8.84 | 7.47 | 1.87E-03 |
| c74593f2p01752 | c74593f2p01752 UDP-arabinose 4-epimerase 1_UDP-glucose 4- | 1754 | -8.91 | 7.52 | 1.87E-03 |
| c60950f1p01620 | c60950f1p01620 sugar transporter ERD6-like 16_vacuolar membrane glucose transmembrane transport | 1628 | 3.4 | -3.64 | 1.87E-03 |
| c44873f1p01424 | c44873f1p01424 beta-hexosaminidase 1_vacuole cytosol | 1444 | -5.03 | 4.22 | 1.87E-03 |

**Table S20. DEGs in high sugar top vs high sugar bottom experiment with SAS database**

| Feature ID | Description | Size | Fold Change (original values) | Weighted proportions fold change | FDR p-value correction |
| --- | --- | --- | --- | --- | --- |
| SCEZLR1031D07.g | sucrose-phosphatase 2 | 497 | 2.17 | 2.46 | 6.51E-03 |
| SCACSB1117F03.g | sucrose synthase | 672 | 1.24 | 4.35 | 4.07E-03 |
| SCEZSD1079C10.g | bidirectional sugar transporter SWEET16-like | 528 | 1.12 | 4.83 | 1.87E-04 |
| SCJLHR1025D07.g | bidirectional sugar transporter SWEET3 | 674 | 1.34 | 4.03 | 7.34E-07 |
| SCRLSD1011H09.b | bidirectional sugar transporter SWEET3 | 564 | 1.47 | 3.68 | 1.32E-03 |
| SCJFRT1010B12.g | probable sugar phosphate phosphate translocator At5g25400 | 630 | 2.43 | 2.22 | 3.49E-08 |

**Table S21. DEGs in low sugar top vs low sugar bottom experiment with SAS database**

| Feature ID | Description | size | Fold Change (original values) | Weighted proportions fold change | FDR p-value correction |
| --- | --- | --- | --- | --- | --- |
| SCACSB1117F03.g | sucrose synthase | 672 | -13.82 | 11.65 | 0.01 |
| SCCCRZ1002G07.g | probable sucrose-phosphate synthase 1 | 3602 | -13.59 | 11.22 | 9.84E-03 |
| SCEQRT2090C11.g | Sucrose transport SUC3 | 1329 | -12.97 | 10.66 | 5.05E-04 |
| SCSBAM1084D02.g | sucrose transport SUC3 isoform X1 | 675 | -9.09 | 7.44 | 7.26E-03 |
| SCCCLR1001A05.g | sucrose synthase | 2737 | -8.51 | 7.04 | 4.67E-04 |
| SCEZLR1052C03.g | sucrose synthase | 877 | -8.37 | 6.91 | 6.84E-04 |
| SCEPAM2014B12.g | sucrose transport SUC3 isoform X2 | 1573 | -7.69 | 6.42 | 1.43E-03 |
| SCEZRZ3099D06.g | sucrose synthase 1 | 927 | -7.51 | 6.31 | 8.54E-04 |
| SCJFST1011B06.g | sucrose nonfermenting 4 | 1250 | -6.39 | 5.4 | 3.39E-04 |
| SCCCRT2001F10.g | sucrose nonfermenting 4 | 1595 | -6.15 | 5.11 | 3.42E-04 |
| SCEZRZ1013G04.g | probable galactinol--sucrose galactosyltransferase 2 | 1937 | -6.09 | 5.04 | 1.07E-03 |
| SCEPLR1008A12.g | sucrose transport SUC4-like | 1964 | -5.94 | 4.94 | 2.87E-04 |
| SCCCLR1C06G07.g | probable galactinol--sucrose galactosyltransferase 1 | 678 | -5.9 | 4.89 | 3.48E-04 |
| SCCCRZ1004G04.g | impaired sucrose induction 1 | 1055 | -5.76 | 4.83 | 5.49E-04 |
| SCRLLR1038B01.g | sucrose nonfermenting 4 | 1565 | -5.71 | 4.81 | 4.83E-04 |
| SCEZLR1031D07.g | sucrose-phosphatase 2 | 497 | -5.59 | 4.7 | 2.62E-04 |
| SCAGRT2037G07.g | sucrose synthase | 3056 | -5.38 | 4.47 | 7.70E-04 |
| SCSBST3096E12.g | sucrose-phosphatase 2-like | 763 | -5.14 | 4.33 | 1.96E-04 |
| SCEZLR1052H03.g | probable galactinol--sucrose galactosyltransferase 2 | 1088 | -5.14 | 4.2 | 0.01 |
| SCUTAM2087H12.g | sucrose-phosphate synthase | 1873 | -5.03 | 4.17 | 3.33E-04 |
| SCACLR1036G09.g | sucrose-phosphate synthase | 800 | -4.92 | 4.08 | 5.63E-04 |
| SCEPCL6023F02.g | sucrose synthase 2 | 2174 | -4.89 | 4.06 | 9.76E-04 |
| SCUTSB1033G12.g | probable galactinol--sucrose galactosyltransferase 2 | 596 | -4.85 | 4.1 | 1.53E-03 |
| SCACAD1036B04.g | probable sucrose-phosphate synthase 1 | 580 | -4.8 | 3.98 | 1.41E-03 |
| SCEQAM1036A06.g | probable sucrose-phosphate synthase 3 | 2030 | -4.41 | 3.74 | 0.01 |
| SCCCAD1002G07.g | sucrose-phosphate synthase | 1265 | -4.3 | 3.59 | 4.19E-03 |
| SCSBLB1032H06.g | probable galactinol--sucrose galactosyltransferase 2 | 471 | -4.25 | 3.55 | 0.01 |
| SCJLHR1025D07.g | bidirectional sugar transporter SWEET3 | 674 | -14.92 | 12.32 | 9.46E-03 |
| SCQGST3153F06.g | sugar transport 5-like | 1899 | -9.65 | 8.24 | 1.12E-03 |
| SCUTFL1058E04.g | sugar phosphatase -like | 502 | -9.61 | 8.13 | 5.62E-03 |
| SCCCLR2C03H09.g | sugar transporter ERD6-like 6 | 823 | -9.36 | 7.91 | 1.34E-03 |
| SCEZAM2031D12.g | UDP-sugar pyrophosphorylase | 758 | -9 | 7.61 | 3.23E-04 |
| SCEQLB1067F03.g | Nucleotide-diphospho-sugar transferase family | 565 | -8.83 | 7.58 | 0.01 |
| SCAGLR1021A01.g | probable sugar phosphate phosphate translocator At5g25400 | 1714 | -8.78 | 7.42 | 3.46E-04 |
| SCJFRT1010B12.g | bidirectional sugar transporter SWEET4-like | 1235 | -8.75 | 7.23 | 2.74E-04 |
| SCJLLB2079F03.g | UDP-sugar pyrophosphorylase | 697 | -8.71 | 7.36 | 9.39E-03 |
| SCBFRZ2018H10.g | UDP-sugar pyrophosphorylase | 677 | -8.38 | 6.95 | 1.91E-03 |
| SCVPFL3046G09.b | sugar transporter ERD6-like 6 | 952 | -7.95 | 6.76 | 8.59E-04 |
| SCEQRT1031C11.g | bidirectional sugar transporter SWEET14-like | 1486 | -7.74 | 6.36 | 0.01 |
| SCEQRT1026B05.g | probable sugar phosphate phosphate translocator At3g11320 | 1268 | -7.46 | 6.3 | 8.79E-04 |
| SCCCLB1004H11.g | bidirectional sugar transporter SWEET1-like | 1362 | -7.33 | 6.1 | 2.59E-03 |
| SCAGFL1088D06.g | probable sugar phosphate phosphate translocator At3g11320 | 835 | -7.3 | 6.06 | 8.56E-04 |
| SCJFLR1017D01.g | probable sugar phosphate phosphate translocator At1g06470 | 1933 | -6.77 | 5.71 | 7.88E-04 |
| SCCCLB1025H10.g | probable sugar phosphate phosphate translocator At3g11320 | 547 | -6.72 | 5.73 | 2.72E-04 |
| SCJFRZ2015H09.g | bidirectional sugar transporter SWEET4-like | 1230 | -6.7 | 5.59 | 6.39E-04 |
| SCJFRZ2015B09.g | probable sugar phosphate phosphate translocator At1g06470 | 665 | -6.56 | 5.44 | 2.02E-03 |
| SCCCLB2001A03.g | Sugar transporter ERD6-like 6 | 622 | -6.52 | 5.5 | 9.39E-04 |
| SCJLRZ1021E01.g | bidirectional sugar transporter SWEET4-like | 1268 | -6.47 | 5.37 | 4.55E-04 |
| SCAGFL1091C07.g | sugar phosphatase | 940 | -6.39 | 5.32 | 3.01E-04 |
| SCSFSB1068F11.g | probable sugar phosphate phosphate translocator At3g14410 | 639 | -6.26 | 5.31 | 3.30E-04 |
| SCBGSD2049G08.g | sugar transport 7 | 864 | -6.25 | 5.24 | 6.77E-03 |
| SCSGRT2065C08.g | sugar transport 5-like isoform X1 | 1229 | -6.17 | 5.16 | 4.28E-03 |
| SCQSRT2032A08.g | sugar transporter ERD6-like 6 | 1410 | -5.77 | 4.82 | 2.62E-03 |
| SCEQAD1015F03.g | probable sugar phosphate phosphate translocator At5g25400 | 1618 | -5.71 | 4.87 | 1.83E-03 |
| SCSGLR1084E11.g | Sugar phosphatase | 660 | -5.65 | 4.81 | 2.64E-03 |
| SCRLLR1016H01.g | Sugar transport 5 | 805 | -5.63 | 4.79 | 3.83E-04 |
| SCCCAD1002A04.g | probable sugar phosphate phosphate translocator At3g17430 | 884 | -5.54 | 4.6 | 7.98E-03 |
| SCQGST1029B12.g | probable sugar phosphate phosphate translocator At5g25400 | 630 | -5.53 | 4.66 | 3.81E-03 |
| SCEQLB1067E10.g | Sugar transporter ERD6-like 6 | 2474 | -5.22 | 4.41 | 3.94E-04 |
| SCQGLR1041F07.g | sugar transporter ERD6-like 5 | 1708 | -5.2 | 4.32 | 4.97E-04 |
| SCCCLR1C02C08.g | bidirectional sugar transporter SWEET2a-like | 772 | -5.12 | 4.35 | 3.63E-04 |
| SCSGHR1068D07.g | UDP-sugar transporter DDB_G0278631 | 1421 | -4.91 | 4.15 | 5.61E-04 |
| SCAGLR1021G05.g | UDP-sugar transporter DDB_G0278631 | 1317 | -3.22 | 2.67 | 6.18E-03 |

**Table S22. DEGs in high sugar bottom vs low sugar bottom experiment with SAS database**

| Feature ID | Description | Size | Fold Change (original values) | Weighted proportions fold change | FDR p-value correction |
| --- | --- | --- | --- | --- | --- |
| SCEQAM1036A06.g | probable sucrose-phosphate synthase 3 | 2030 | -7.47 | 6.29 | 4.37E-06 |
| SCEZLR1052H03.g | probable galactinol--sucrose galactosyltransferase 2 | 1088 | -7.08 | 5.84 | 7.54E-04 |
| SCEPAM2014B12.g | sucrose transport SUC3 isoform X2 | 1573 | -7.08 | 5.94 | 2.03E-05 |
| SCEPCL6023F02.g | sucrose synthase 2 | 2174 | -6.89 | 5.72 | 2.30E-04 |
| SCSBLB1032H06.g | probable galactinol--sucrose galactosyltransferase 2 | 471 | -6.78 | 5.66 | 3.39E-06 |
| SCCCRT2001F10.g | sucrose nonfermenting 4 | 1595 | -6.08 | 5.07 | 1.81E-06 |
| SCJFST1011B06.g | sucrose nonfermenting 4 | 1250 | -5.88 | 4.87 | 1.39E-05 |
| SCRLLR1038B01.g | sucrose nonfermenting 4 | 1565 | -5.77 | 4.82 | 2.75E-06 |
| SCCCLR1C06G07.g | probable sucrose-phosphate synthase 1 | 3602 | -5.63 | 4.64 | 4.97E-06 |
| SCEQRT2090C11.g | Sucrose transport SUC3 | 1329 | -5.51 | 4.56 | 2.12E-05 |
| SCSBST3096E12.g | sucrose-phosphatase 2-like | 763 | -5.21 | 4.36 | 3.55E-05 |
| SCCCRZ1004G04.g | impaired sucrose induction 1 | 1055 | -5.21 | 4.36 | 7.47E-05 |
| SCACAD1036B04.g | probable sucrose-phosphate synthase 1 | 580 | -5.17 | 4.3 | 2.18E-06 |
| SCEZRZ1013G04.g | probable galactinol--sucrose galactosyltransferase 2 | 1937 | -5.11 | 4.26 | 1.33E-05 |
| SCUTAM2087H12.g | sucrose-phosphate synthase | 1873 | -5.06 | 4.19 | 2.42E-06 |
| SCAGRT2037G07.g | sucrose-phosphate synthase | 1265 | -4.85 | 4.04 | 4.83E-05 |
| SCEPLR1008A12.g | sucrose transport SUC4-like | 1964 | -4.81 | 4 | 4.82E-06 |
| SCACLR1036G09.g | sucrose-phosphate synthase | 800 | -4.6 | 3.83 | 6.33E-06 |
| SCEZLR1052C03.g | sucrose synthase | 877 | -4.56 | 3.77 | 1.72E-03 |
| SCCCLR1001A05.g | sucrose synthase | 2737 | -4.45 | 3.68 | 4.39E-04 |
| SCEZLR1031D07.g | sucrose-phosphatase 2 | 497 | -4.34 | 3.66 | 3.66E-06 |
| SCCCAD1002G07.g | probable galactinol--sucrose galactosyltransferase 1 | 678 | -3.87 | 3.24 | 8.29E-03 |
| SCUTSB1033G12.g | probable galactinol--sucrose galactosyltransferase 2 | 596 | -3.81 | 3.15 | 2.72E-03 |
| SCUTFL1058E04.g | sugar phosphatase -like | 502 | -7.8 | 6.64 | 6.02E-04 |
| SCCCLR2C03H09.g | bidirectional sugar transporter SWEET4-like | 1230 | -7.35 | 6.19 | 6.07E-03 |
| SCEZAM2031D12.g | UDP-sugar pyrophosphorylase | 758 | -7.29 | 6.09 | 2.74E-04 |
| SCEQRT1031C11.g | bidirectional sugar transporter SWEET14-like | 1486 | -7.17 | 5.83 | 2.06E-03 |
| SCBGSD2049G08.g | sugar transport 7 | 864 | -6.72 | 5.62 | 8.51E-05 |
| SCSGRT2065C08.g | bidirectional sugar transporter SWEET2a-like | 772 | -6.37 | 5.24 | 2.00E-04 |
| SCAGLR1021A01.g | probable sugar phosphate phosphate translocator At5g25400 | 1714 | -6.34 | 5.26 | 9.12E-05 |
| SCJFRT1010B12.g | probable sugar phosphate phosphate translocator At5g25400 | 630 | -6.32 | 5.23 | 1.04E-05 |
| SCVPFL3046G09.b | sugar transporter ERD6-like 6 | 952 | -6.28 | 5.31 | 0.01 |
| SCAGFL1088D06.g | probable sugar phosphate phosphate translocator At3g11320 | 835 | -6.25 | 5.21 | 0.01 |
| SCJFLR1017D01.g | probable sugar phosphate phosphate translocator At1g06470 | 1933 | -5.87 | 4.97 | 5.64E-05 |
| SCRLLR1016H01.g | Sugar transport 5 | 805 | -5.87 | 4.93 | 3.91E-04 |
| SCSFSB1068F11.g | probable sugar phosphate phosphate translocator At3g14410 | 639 | -5.82 | 4.91 | 3.60E-05 |
| SCJFRZ2015H09.g | bidirectional sugar transporter SWEET4-like | 1230 | -5.67 | 4.76 | 2.32E-06 |
| SCEQRT1026B05.g | probable sugar phosphate phosphate translocator At3g11320 | 1268 | -5.6 | 4.66 | 7.73E-05 |
| SCAGFL1091C07.g | sugar phosphatase | 940 | -5.56 | 4.6 | 1.14E-05 |
| SCCCAD1002A04.g | probable sugar phosphate phosphate translocator At3g17430 | 884 | -5.55 | 4.56 | 7.64E-03 |
| SCRLSD1011H09.b | bidirectional sugar transporter SWEET16-like | 528 | -5.52 | 4.62 | 3.11E-03 |
| SCCCLB1025H10.g | UDP-sugar pyrophosphorylase | 677 | -5.44 | 4.55 | 2.31E-05 |
| SCSGLR1084E11.g | Sugar phosphatase | 660 | -5.43 | 4.51 | 2.37E-03 |
| SCEQLB1067E10.g | Sugar transporter ERD6-like 6 | 2474 | -5.34 | 4.51 | 2.33E-06 |
| SCJLRZ1021E01.g | bidirectional sugar transporter SWEET4-like | 1268 | -5.33 | 4.38 | 1.31E-03 |
| SCQSRT2032A08.g | sugar transporter ERD6-like 6 | 1410 | -5.32 | 4.41 | 4.52E-03 |
| SCSGHR1068D07.g | UDP-sugar transporter DDB_G0278631 | 1421 | -5.22 | 4.39 | 7.10E-05 |
| SCQGST1029B12.g | probable sugar phosphate phosphate translocator At5g25400 | 630 | -5.13 | 4.27 | 8.08E-03 |
| SCCCLR1C02C08.g | bidirectional sugar transporter SWEET2a-like | 772 | -5.08 | 4.27 | 2.09E-04 |
| SCCCLB2001A03.g | Sugar transporter ERD6-like 6 | 622 | -5.08 | 4.24 | 6.89E-05 |
| SCAGLR1021G05.g | UDP-sugar transporter DDB_G0278631 | 1317 | -5.07 | 4.2 | 8.33E-06 |
| SCQGLR1041F07.g | sugar transporter ERD6-like 5 | 1708 | -4.8 | 3.99 | 3.11E-06 |
| SCEQAD1015F03.g | Nucleotide-diphospho-sugar transferase family | 565 | -4.28 | 3.56 | 0.01 |
